# CDCA8 regulates ATP5F1A protein stability and malignant phenotypes in wilms tumor cells: Prognostic implications and mechanistic insights

**DOI:** 10.1371/journal.pone.0353696

**Published:** 2026-07-21

**Authors:** Qiang Zeng, Junfeng Tao, Guangbei Peng, Lilu Qin, Shuzhen Wu, Chen Wang, Zhong Liu, Linshan Zeng

**Affiliations:** 1 Department of Pediatric Surgery, Jiangxi Maternal and Child Health Hospital, Jiangxi Children’s Medical Center, Nanchang, Jiangxi, P. R. China; 2 Department of Pediatric Surgery, The First Affiliated Hospital of Gannan Medical University, Ganzhou, Jiangxi, P. R. China; Washington State University, UNITED STATES OF AMERICA

## Abstract

**Background:**

Wilms tumor (WT) is a prevalent pediatric renal malignancy, yet its molecular mechanisms remain poorly defined. Identifying key prognostic genes and understanding their functional roles is critical for improving clinical management. This study aimed to identify prognostic genes in WT and to investigate whether ATP5F1A functions as a candidate downstream effector in a CDCA8-associated regulatory axis.

**Methods:**

Differentially expressed genes (DEGs) were identified by analyzing RNA-seq datasets (GSE11151, GSE73209), comparing WT tissues with normal kidney samples. Functional enrichment was performed using Gene Ontology (GO) and Kyoto Encyclopedia of Genes and Genomes (KEGG) analyses. A protein-protein interaction (PPI) network was constructed, and hub genes were identified using Cytoscape. Prognostic significance was assessed through univariate and multivariate Cox regression analyses, and a risk prediction model was developed. External expression validation of candidate hub genes was performed using GSE11024 and GSE110696. CDCA8’s functional role in WT was validated by gene knockdown and overexpression experiments. Co-immunoprecipitation (Co-IP) and ubiquitination assays were conducted to explore the interaction between CDCA8 and ATP5F1A.

**Results:**

A total of 987 DEGs were identified, with six hub genes—BUB1B, CDC45, CDCA8, KIF15, NDC80, and TOP2A—were identified. The exploratory risk score model stratified overall survival in the TARGET-WT cohort (HR = 2.973, Log-rank p = 0.000252), although time-dependent AUC values indicated modest discriminatory performance. CDCA8 was upregulated in WT tissues and associated with worse survival outcomes (HR = 2.03, p = 0.011). Functional assays suggested that CDCA8 knockdown suppressed malignant phenotypes in WiT49 cells. Mechanistically, CDCA8 knockdown reduced ATP5F1A protein levels without altering ATP5F1A mRNA expression, and this reduction was partially rescued by MG132. CDCA8 depletion was also associated with increased ATP5F1A ubiquitination, supporting a role for CDCA8 in maintaining ATP5F1A protein stability.

**Conclusions:**

These findings suggest that CDCA8 may contribute to Wilms tumor cell phenotypes partly by maintaining ATP5F1A protein stability, providing preliminary evidence for a CDCA8-ATP5F1A regulatory axis in WT.

## Introduction

Wilms tumor (WT) is a pediatric embryonal malignancy that represents a unique intersection between disruptions in organogenesis and tumorigenesis. It is closely associated with early kidney development and shares morphological similarities with this process [1]. WT represents the most common pediatric renal malignancy and accounts for the majority of childhood kidney tumors, while also comprising a notable proportion of pediatric cancers [2–4]. Despite substantial progress in treatment, WT remains a leading cause of cancer-related morbidity and mortality in children, particularly in cases of advanced disease or resistance to conventional therapy. In up to 15% of WT cases, malignant progression is associated with germline mutations in high-risk cancer genes, with about one-third of patients exhibiting genetic alterations in WT1, Ctnnb1, and Wtx [5,6]. These genetic factors highlight the complex molecular landscape of WT and underscore the need for more effective prognostic tools and targeted therapies.

The treatment of WT remains challenging due to the disease’s heterogeneity and the lack of reliable biomarkers for early detection and prognosis. Current therapeutic strategies primarily rely on tumor staging and histological classification; however, clinical outcomes remain suboptimal for high-risk patients. Personalized treatment, based on the genetic and molecular profile of the tumor, is critical to improving patient survival while reducing unnecessary treatment-related toxicity. Stratifying treatment approaches according to the molecular characteristics of the tumor could provide more tailored therapies, potentially enhancing outcomes and minimizing overtreatment.

Recent studies have underscored the importance of genetic alterations in the pathogenesis of WT [1,3]. These genetic mutations and disruptions in signaling pathways not only drive tumor initiation but also affect the response to therapy [7,8]. This makes genetic profiling a key strategy for identifying individuals at high risk of developing WT and for personalizing therapeutic regimens. Understanding the molecular mechanisms driving WT may provide insight into disease biology and help identify candidate mechanisms for future investigation

CDCA8, a gene involved in cell cycle regulation, has been implicated in the progression of various cancers [9–11], but its role in Wilms tumor has not been thoroughly explored. Investigating the expression and function of CDCA8 in WT may provide insight into WT-associated cell-cycle dysregulation and identify candidate molecular mechanisms for further investigation. Additionally, analyzing differentially expressed genes (DEGs) and their correlation with clinical outcomes could further elucidate the genetic landscape of WT, offering new biomarkers for early detection and prognosis.

ATP5F1A encodes the alpha subunit of mitochondrial ATP synthase and participates in oxidative phosphorylation and mitochondrial energy metabolism [12]. Mitochondrial metabolism and oxidative phosphorylation are increasingly recognized as important contributors to tumor progression, including in pediatric malignancies. Therefore, ATP5F1A may represent a biologically relevant molecule linking cell-cycle dysregulation and metabolic adaptation in WT. In the present study, ATP5F1A was not selected as an initial prognostic candidate but emerged from PPI-based screening as a potential CDCA8-associated protein and was subsequently investigated through experimental validation.

In this study, we integrated transcriptomic data from the Therapeutically Applicable Research to Generate Effective Treatments (TARGET) database and Gene Expression Omnibus (GEO) datasets to identify differentially expressed genes, candidate hub genes, and molecular pathways associated with WT. We further evaluated the prognostic relevance of selected hub genes and prioritized CDCA8 for functional validation based on its elevated expression in WT and its association with adverse clinical outcomes. Using WiT49 cells, we investigated the effects of CDCA8 knockdown on malignant cellular phenotypes and explored whether CDCA8 may regulate ATP5F1A protein stability. Overall, this study aimed to provide preliminary insight into the prognostic significance and potential functional role of CDCA8 in WT, while identifying a candidate CDCA8-ATP5F1A regulatory axis that warrants further validation.

## Methods

### Data acquisition and preprocessing

Transcriptomic data and corresponding clinical information for Wilms tumor were obtained from the Therapeutically Applicable Research to Generate Effective Treatments-Wilms Tumor cohort (TARGET-WT). In addition, four microarray expression datasets, GSE11151, GSE73209, GSE11024, and GSE110696, were downloaded from the NCBI Gene Expression Omnibus database. The platform information, sample size, and specific use of each dataset are summarized in Table 1. GSE11151 and GSE73209 were used as discovery datasets for DEG identification, whereas GSE11024 and GSE110696 were used as external validation datasets. TARGET-WT was used for histological subgroup comparison and prognostic model construction.

**Table 1:** Characteristics of the included datasets.

Dataset	Country	Platforms	No. of samples	Usage here
GSE11151	Netherlands	GPL570	Wilms tumor(n = 4), Normal kidney (n = 5)	Identification of hub genes
GSE73209	Sweden	GPL10558	Wilms tumor(n = 32), Normal kidney (n = 6)	Identification of hub genes
GSE11024	USA	GPL6671	Wilms tumor(n = 27), Normal kidney(n = 12)	Verification of hub genes
GSE110696	USA	GPL23126	Wilms tumor(n = 13), Normal kidney(n = 6)	Verification of hub genes
TARGET-WT	USA	RNA seq	DAWT(n = 22), FHWT(n = 114)	Verification of hub genes, Construction and identification of the prognostic model

For GEO datasets, normalized series matrix files were downloaded when available. Expression values were log2-transformed when required. Probe identifiers were mapped to official gene symbols according to the corresponding platform annotation files. When multiple probes mapped to the same gene symbol, the average expression value was used for downstream analysis. Differential expression analysis was performed independently in GSE11151 and GSE73209 using the limma package in R. Genes with an adjusted p-value < 0.05 and an absolute log2 fold change > 1.3 were defined as differentially expressed genes. The Benjamini-Hochberg method was used to control the false discovery rate. To reduce platform-specific bias, DEGs were identified separately in the two discovery datasets, and overlapping upregulated and downregulated genes were retained for subsequent analyses.

For TARGET-WT RNA-seq data, expression values were processed as FPKM. Clinical information, including survival time, survival status, and histological classification, was matched with expression data according to patient identifiers. Samples with missing survival information were excluded from survival analyses.

### Gene ontology (GO) and kyoto encyclopedia of genes and genomes (KEGG) pathway enrichment analysis

Pathway enrichment analysis for the identified differentially expressed genes (DEGs) was performed using the Database for Annotation, Visualization, and Integrated Discovery (DAVID) [13]. Both Gene Ontology (GO) and Kyoto Encyclopedia of Genes and Genomes (KEGG) enrichment analyses were conducted to investigate the biological pathways and functional implications of the DEGs. Statistical significance was defined as p < 0.05. The GO enrichment analysis was carried out across three major categories: Biological Processes (BP), Molecular Functions (MF), and Cellular Components (CC). A minimum gene count of 10 was applied as a filtering threshold for each GO term. Reference gene sets for the BP, MF, and CC categories were obtained from the Molecular Signatures Database (MSigDB) [14].

### Protein-protein interaction (PPI) network analysis

A Protein-Protein Interaction (PPI) network for the differentially expressed genes (DEGs) was constructed using the Search Tool for the Retrieval of Interacting Genes/Proteins (STRING) database (http://string-db.org) with a confidence score ≥ 0.400 [15]. The PPI data were subsequently imported into Cytoscape 3.9.2 (http://cytoscape.org/) for network visualization and further analysis [16,17] Central hub genes were identified using the default settings with the Degree, Maximum Clique Centrality (MCC), Maximum Neighborhood Component (MNC), and Molecular Complex Detection (MCODE) algorithms.

To identify candidate CDCA8-associated proteins for mechanistic validation, a CDCA8-centered PPI network was further constructed using STRING. Candidate interacting proteins were ranked based on STRING interaction evidence and biological relevance. ATP5F1A was selected for further experimental validation as a candidate CDCA8-associated protein.

### ATP5F1A expression and correlation analysis

To strengthen the connection between the transcriptomic analysis and mechanistic validation, ATP5F1A expression and its correlation with CDCA8 were further evaluated in available WT datasets. In the four GEO datasets, including GSE11151, GSE73209, GSE11024, and GSE110696, ATP5F1A expression was compared between Wilms tumor tissues and normal kidney tissues. In the TARGET-WT cohort, ATP5F1A expression was compared between DAWT and FHWT. Differences in ATP5F1A expression between two groups were assessed using the Wilcoxon rank-sum test.

The correlation between CDCA8 and ATP5F1A expression was analyzed separately in each GEO dataset using all included samples, including both Wilms tumor and normal kidney tissues. In the TARGET-WT cohort, which contained tumor samples only, the relationship between CDCA8 and ATP5F1A expression was assessed across all available WT tumor samples without stratification by histological subtype. Spearman correlation analysis was used to assess the association between CDCA8 and ATP5F1A expression.

These analyses were performed to determine whether ATP5F1A expression differed across WT-related clinical or tissue groups and whether ATP5F1A was associated with CDCA8 at the transcriptomic level, thereby providing additional bioinformatic support for subsequent mechanistic validation.

### Survival analysis and construction of the prognostic risk model

Survival analysis was performed to evaluate the prognostic significance of the candidate hub genes in Wilms tumor. The expression matrix of the six candidate hub genes was integrated with overall survival time and survival status from the TARGET-WT cohort. Patients with missing survival information were excluded from the survival analysis.

For each hub gene, patients were stratified into high- and low-expression groups according to the median expression value. Kaplan-Meier survival curves were generated to compare overall survival between the two groups, and p-values were calculated using the log-rank test. Hazard ratios (HRs) and 95% confidence intervals (CIs) were estimated using univariate Cox proportional hazards regression analysis. Because six hub genes were tested simultaneously, p-values were adjusted using the Benjamini-Hochberg false discovery rate method.

To construct a prognostic risk model, a multivariable Cox regression model was first established using the six candidate hub genes. The risk score for each patient was calculated as follows: risk score = Σ βi × Expi, where βi represents the regression coefficient of each gene derived from the multivariable Cox model, and Expi represents the expression value of the corresponding gene. Patients were divided into high-risk and low-risk groups according to the median risk score. Kaplan-Meier survival analysis was then performed to compare overall survival between the high-risk and low-risk groups, with statistical significance assessed using the log-rank test. HRs and 95% CIs were estimated using univariate Cox regression.

To identify the optimal reduced prognostic model, stepwise Cox regression analysis was performed using the step function in R after initial multivariable Cox regression analysis. The model selected by iterative stepwise regression was used as the final reduced model. Time-dependent receiver operating characteristic curves were generated to evaluate the predictive performance of the model at 1, 3, 5, and 10 years. All statistical analyses were performed using R software version 4.0.3. All statistical tests were two-sided, and p-values<0.05 were considered statistically significant.

### Cell culture

HEK 293T cells were obtained from the American Type Culture Collection (ATCC, USA). WiT49 cells were used as the primary WT cell model, as described previously (PMID: 39659787, PMID: 38937666) [16]. HEK 293T cells were used for lentiviral packaging and were not considered an additional WT model. Cells were routinely tested for mycoplasma contamination using a Mycoplasma Detection Kit (Solarbio, CA1080, China) according to the manufacturer’s instructions, and only mycoplasma-negative cells were used in this study.

### shRNA and overexpression assay

Target-specific short hairpin RNA (shRNA) sequences against CDCA8 were designed and cloned into the pLKO-puro lentiviral vector. A non-targeting shRNA was used as the negative control. For lentivirus production, the shRNA plasmid was co-transfected with the packaging plasmids psPAX2 and pVSV-G into HEK 293T cells using Lipofectamine 2000 (Invitrogen, USA) according to the manufacturer’s instructions. Viral supernatants were collected 72 h after transfection, filtered through a 0.45 μm filter, and used to infect WiT49 cells. The cDNAs of ATP5F1A were cloned into the HA-pcDNA3.0 vector for overexpression.Stable knockdown or overexpression cells were selected with puromycin according to the optimized selection conditions for WiT49 cells.nThe specific shRNA sequences and overexpression constructs used in the study are listed in Table 2.

**Table 2:** ShRNA Sequences and Overexpression Constructs Used in This Study.

Gene	Target sequence
sh NC	TTCTCCGAACGTGTCACGT
sh CDCA8−1	CGCCTCCTTTCTGAAAGACTT
sh CDCA8−2	GTTTGACTCAAGGGTCTTCAA
OE ATP5F1A	NC_000018.10

### Western blotting

Total proteins were extracted from WiT49 and HEK 293T cells for analysis. Protein concentrations were quantified using the BCA assay. 25 μg of protein from each sample were resolved by SDS-PAGE and transferred to 0.22 μm PVDF membranes. Membranes were blocked for 1 hour using a blocking solution. After blocking, the membranes were incubated overnight at 4°C with primary antibodies, followed by incubation with the secondary antibody at room temperature for 1 hour. The blots were then imaged. The following primary antibodies were used: Anti-CDCA8 (1:1000 dilution, 12465–1-AP, Proteintech), Anti-ATP5F1A (1:1000 dilution, 14676–1-AP, Proteintech), anti-α-Tubulin (1:5000 dilution, 11224–1-AP, Proteintech), and anti-Ubiquitin (P4D1) (1:1000 dilution, sc-8017, Santa Cruz).

### Reverse transcription-polymerase chain reaction (RT-PCR)

Cells subjected to various experimental conditions were used for RNA extraction and cDNA synthesis, as previously described [16]. All RT reactions were performed simultaneously to ensure consistency across experiments, with 10 ng of RNA per reaction. Each sample was tested in triplicate wells. The primers used in this study are listed in Table 3.

**Table 3:** Primers used for quantitative real time PCR.

Oligonucleotides	Sequence (5′–3′)
GAPDH-F	GGAGCGAGATCCCTCCAAAAT
GAPDH-R	GGCTGTTGTCATACTTCTCATGG
CDCA8-F	GCAGGAGAGCGGATTTACAAC
CDCA8-R	CTGGGCAATACTGTGCCTCTG
ATP5F1A-F	GCTCCTTACTCTGGCTGTTCCA
ATP5F1A-R	GCGGAGCAACAGAGACATCTGA

### Cell viability and colony formation assay

Cell viability was assessed using the Cell Counting Kit-8 (CCK-8; Dojindo, Japan). Briefly, transfected WiT49 cells were seeded into 96-well plates at a density of 3 × 10^3^ cells per well. At 24, 48, and 72 h after seeding, 10 μl of CCK-8 reagent was added to each well, and the cells were incubated for an additional 2 h at 37°C. Absorbance at 450 nm was then measured using a microplate reader. Each experimental condition was assessed in technical triplicate, and the assay was repeated in three independent biological replicates.

For the colony formation assay, 1000 transfected WiT49 cells were seeded into each well of 6-well plates and cultured for 14 days, with the medium changed every 4 days. The colonies were washed three times with phosphate-buffered saline (PBS), fixed with 4% paraformaldehyde, and stained with 1% crystal violet. Colonies containing more than 50 cells were counted and recorded. The experiment was repeated in three independent biological replicates.

### Cell migration and invasion assays

Cell migration and invasion assays were performed using transwell chambers. For the migration assay, transfected WiT49 cells were resuspended in serum-free medium and seeded into the upper chambers without Matrigel coating. For the invasion assay, the upper chambers were precoated with Matrigel (BD Biosciences, USA) before cell seeding. A total of 2 × 10^4^ cells were added to each upper chamber, and 700 μl of medium containing 10% FBS was added to the lower chamber as a chemoattractant.

After 48 h of incubation, cells remaining on the upper surface of the membrane were gently removed. Cells that had migrated or invaded to the lower surface of the membrane were fixed with 4% paraformaldehyde for 20 min and stained with 0.1% crystal violet for 30 min. After washing three times with PBS, migrated or invaded cells were observed and counted under a phase-contrast microscope (Nikon, Japan). Cells were counted in five randomly selected fields per membrane. Each experiment was repeated in three independent biological replicates.

### Co-immunoprecipitation (Co-IP) analysis

Co-immunoprecipitation (Co-IP) was performed to assess the association between CDCA8 and ATP5F1A. WiT49 cells were lysed in immunoprecipitation lysis buffer supplemented with protease inhibitors, and a fraction of the lysate was retained as input. Equal amounts of protein were incubated overnight at 4°C with anti-CDCA8, anti-ATP5F1A, or normal IgG control antibodies, followed by incubation with protein A/G magnetic beads for 4 h. The beads were washed three times with binding buffer, and bound proteins were eluted with 1 × SDS loading buffer. The immunoprecipitates and input lysates were analyzed by Western blotting.

### Protein degradation inhibitor treatment and cycloheximide chase assay

To evaluate whether ATP5F1A protein reduction was associated with proteolytic degradation, CDCA8 knockdown and control WiT49 cells were treated with MG132, chloroquine, Eeyarestatin I, or leupeptin. ATP5F1A protein levels were then assessed by Western blotting. For cycloheximide chase assays, cells were treated with cycloheximide to block de novo protein synthesis and collected at the indicated time points. Where indicated, cells were co-treated with MG132. ATP5F1A protein levels were quantified by densitometric analysis, normalized to α-Tubulin, and expressed relative to the level at 0 h.

### Statistical analysis

Data analysis and visualization were performed using R software (version 4.0.3) and GraphPad Prism (version 9.0). Continuous variables were compared between two groups using the Wilcoxon rank-sum test. For comparisons among more than two groups, the Kruskal-Wallis test was used. Correlations between CDCA8 and ATP5F1A expression were assessed using Spearman correlation analysis.

Kaplan-Meier survival curves were generated to evaluate overall survival and were compared using the log-rank test. Hazard ratios (HRs) and 95% confidence intervals (CIs) were estimated using Cox proportional hazards regression analysis with the survival package in R. For survival analyses involving multiple hub genes, *p*-values were adjusted using the Benjamini-Hochberg false discovery rate method.

Data from cell-based experiments are presented as the mean ± standard deviation from at least three independent biological replicates unless otherwise indicated. All statistical tests were two-sided, and *p* < 0.05 was considered statistically significant. Significance levels were defined as *p < 0.05, **p < 0.01, and ***p < 0.001.

## Results

### Identification of differentially expressed genes in wilms tumor

**Fig 1:**
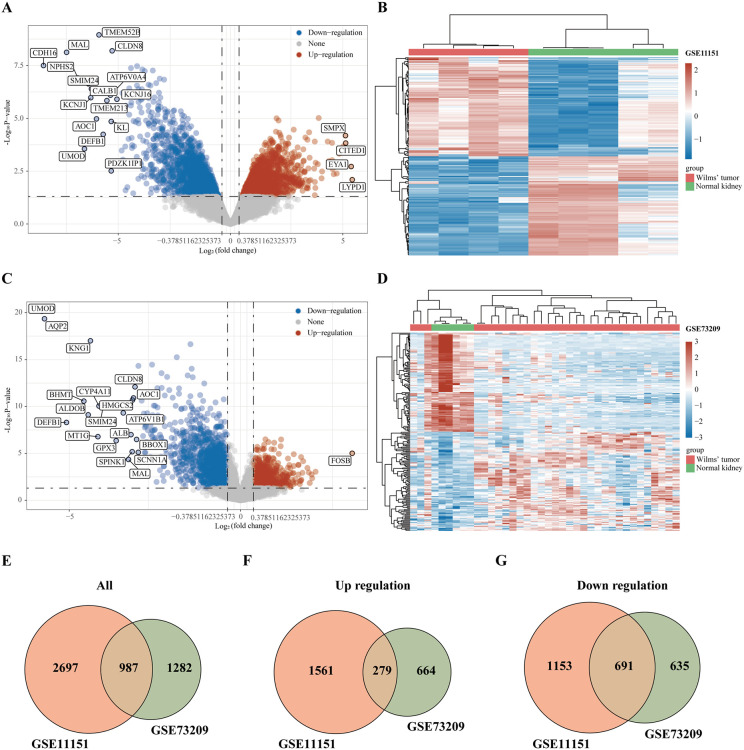
Differentially expressed genes. (A)Volcano plot of DEGs in GSE11151. (B) Heatmap plots of DEGs in GSE11151. (C) Volcano plot of DEGs in GSE73209. (D) Heatmap plots of DEGs in GSE73209. (E) Venn diagram showing overlapping DEGs between GSE11151 and GSE73209. (F) Venn diagram showing commonly upregulated DEGs. (G) Venn diagram showing commonly downregulated DEGs. The green circle represents DEGs identified in GSE73209, and the red circle represents DEGs identified in GSE11151.

To identify differentially expressed genes (DEGs) associated with Wilms tumor (WT), we analyzed two independent GEO datasets, GSE11151 and GSE73209. In GSE11151, which included 4 WT samples and 5 normal kidney samples, 3684 DEGs were identified, including 1840 upregulated and 1844 downregulated genes in WT tissues compared with normal kidney tissues (Fig 1A and 1B). In GSE73209, which included 32 WT samples and 6 fetal normal kidney samples, 3752 DEGs were identified, including 1751 upregulated and 2,001 downregulated genes in WT tissues compared with fetal normal kidney tissues (Fig 1C and 1D).

To identify DEGs consistently altered across datasets, we compared the DEGs obtained from GSE11151 and GSE73209 using Venn analysis. A total of 987 overlapping DEGs were identified between the two datasets (Fig 1E), including 279 commonly upregulated genes (Fig 1F) and 691 commonly downregulated genes (Fig 1G). These overlapping DEGs were used for subsequent functional enrichment and hub gene screening analyses.

### Functional enrichment analyses

To further characterize the biological functions and pathways associated with the overlapping DEGs in Wilms tumor (WT), we performed Gene Ontology (GO) enrichment analysis and Kyoto Encyclopedia of Genes and Genomes (KEGG) pathway analysis.

**Fig 2:**
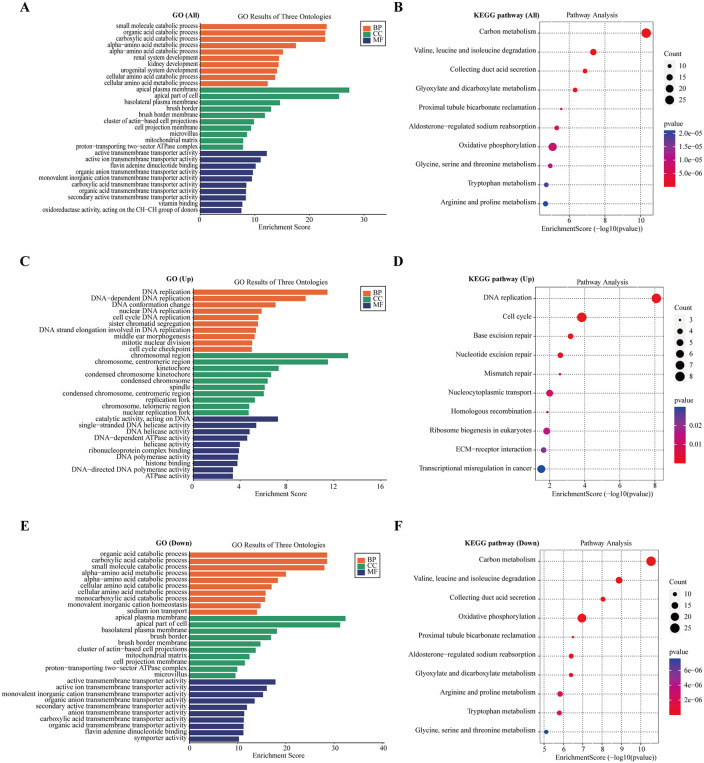
Functional enrichment analysis of the DEGs. (A) GO enrichment of all DEGs. (B) Enriched KEGG pathways of all DEGs. (C) GO enrichment of upregulated DEGs. (D) Enriched KEGG pathways of the upregulated DEGs. (E) GO enrichment of downregulated DEGs. (F) Enriched KEGG pathways of the downregulated DEGs.

The full set of 987 overlapping DEGs was first subjected to enrichment analysis. In the GO Biological Process (GO-BP) category, these genes were mainly enriched in small molecule catabolic process, organic acid catabolic process, and carboxylic acid catabolic process. In the Cellular Component (GO-CC) category, the DEGs were enriched in apical plasma membrane, apical part of cell, and basolateral plasma membrane. In the Molecular Function (GO-MF) category, the DEGs were enriched in active transmembrane transporter activity, active ion transmembrane transporter activity, and flavin adenine dinucleotide binding (Fig 2A). KEGG pathway analysis showed enrichment in carbon metabolism, valine, leucine and isoleucine degradation, and collecting duct acid secretion (Fig 2B), suggesting that metabolic and kidney-related transport processes may be altered in WT.

Enrichment analysis of the 279 commonly upregulated DEGs revealed predominant involvement in cell cycle and DNA replication-related processes. GO-BP terms were mainly enriched in DNA replication, DNA-dependent DNA replication, and DNA conformation change. In the GO-CC category, these genes were enriched in chromosomal region, chromosome centromeric region, and kinetochore, consistent with roles in chromosome organization and mitotic regulation. GO-MF analysis showed enrichment in catalytic activity acting on DNA, single-stranded DNA helicase activity, and DNA helicase activity (Fig 2C). KEGG pathway analysis further identified enrichment in DNA replication, cell cycle, and base excision repair (Fig 2D), supporting the enrichment of proliferative and genome maintenance-related pathways among the upregulated DEGs.

Enrichment analysis of the 691 commonly downregulated DEGs indicated alterations in metabolic and renal epithelial transport-related functions. GO-BP terms were mainly enriched in organic acid catabolic process, carboxylic acid catabolic process, and small molecule catabolic process. The most enriched GO-CC terms included apical plasma membrane, apical part of cell, and basolateral plasma membrane. In the GO-MF category, these genes were enriched in active transmembrane transporter activity, active ion transmembrane transporter activity, and monovalent inorganic cation transmembrane transporter activity (Fig 2E). KEGG pathway analysis identified enrichment in carbon metabolism, valine, leucine and isoleucine degradation, and collecting duct acid secretion (Fig 2F), suggesting reduced representation of metabolic and kidney-specific transport pathways in WT tissues compared with normal kidney tissues.

### Identification and verification of hub genes

**Fig 3:**
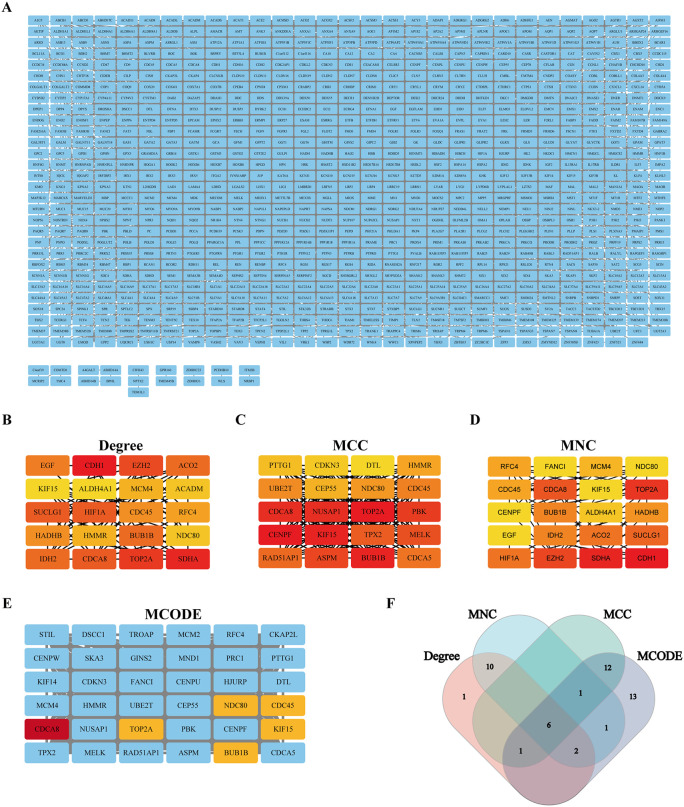
Construction of the PPI network and identification of candidate hub genes. (A) Protein-protein interaction (PPI) network constructed from the 987 overlapping DEGs. (B) Top 20 genes ranked by the Degree algorithm. (C)Top 20 genes ranked by the Maximum Clique Centrality (MCC) algorithm. (D) Top 20 genes ranked by the Maximum Neighborhood Component (MNC) algorithm. (E) The top-ranked module identified by the MCODE algorithm, containing 36 genes with a module score of 33.2. (F) Venn diagram showing the overlapping genes identified by the Degree, MCC, MNC, and MCODE algorithms.

To identify candidate hub genes associated with Wilms tumor (WT), the 987 overlapping DEGs were imported into Cytoscape to construct a protein-protein interaction (PPI) network (Fig 3A). The detailed list of proteins included in the PPI network was provided in S1 Table in S1 File. The top 20 ranked genes were identified using three topological algorithms implemented in Cytoscape version 3.9.2, including Degree (Fig 3B), Maximum Clique Centrality (MCC) (Fig 3C), and Maximum Neighborhood Component (MNC) (Fig 3D). In addition, the MCODE algorithm was used to identify highly connected gene modules within the PPI network. The top-ranked MCODE module was selected based on node connectivity and network density (Fig 3E). The detailed list of proteins included was provided in S2 Table in S1 File.

To further refine hub gene selection, overlap analysis was performed among the genes identified by the Degree, MCC, MNC, and MCODE algorithms using a Venn diagram (Fig 3F). This analysis identified six overlapping candidate hub genes: BUB1B, CDC45, CDCA8, KIF15, NDC80, and TOP2A, which were selected for subsequent expression validation and prognostic analysis.

### Analysis and validation of key hub genes expression in WT patients

**Fig 4:**
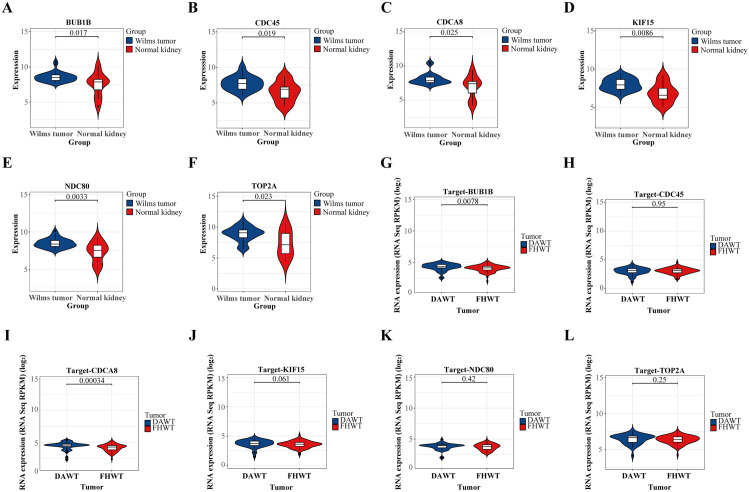
Validation of candidate hub gene expression in WT datasets. (A)-(F) Expression validation of the six candidate hub genes in the combined discovery datasets GSE11151 and GSE73209. (G)-(L) Differential expression of the six candidate hub genes between difficult-to-treat Wilms tumor (DAWT) and favorable histology Wilms tumor (FHWT) samples in the TARGET-WT cohort.

To validate the expression levels of the six candidate hub genes identified in Wilms tumor, their expression patterns were first evaluated in GSE11151 and GSE73209, which together included 36 WT samples and 11 normal kidney samples. The results showed that BUB1B, CDC45, CDCA8, KIF15, NDC80, and TOP2A were all significantly upregulated in WT tissues compared with normal kidney tissues (Figs 4A–F).

Wilms tumor can be classified into clinically distinct histological categories, including difficult-to-treat Wilms tumor (DAWT) and favorable histology Wilms tumor (FHWT). DAWT generally represents a more aggressive subtype with poorer clinical outcomes, whereas FHWT is associated with a more favorable prognosis. To further evaluate the clinical relevance of the six hub genes, their expression levels were analyzed in DAWT and FHWT samples from the TARGET-WT cohort. BUB1B (Fig 4G) and CDCA8 (Fig 4I) were significantly upregulated in DAWT compared with FHWT (p < 0.05), whereas CDC45 (Fig 4H), KIF15 (Fig 4J), NDC80 (Fig 4K), and TOP2A (Fig 4L) showed no significant differences between the two groups (p > 0.05).

These results suggest that BUB1B and CDCA8 may be associated with the more aggressive DAWT phenotype. Consequently, these genes may serve as potential biomarkers for prognostic stratification and potential therapeutic targeting in WT patients.

### Identification and validation of prognostic hub genes and construction of a risk prediction model in wilms tumor patients

**Fig 5:**
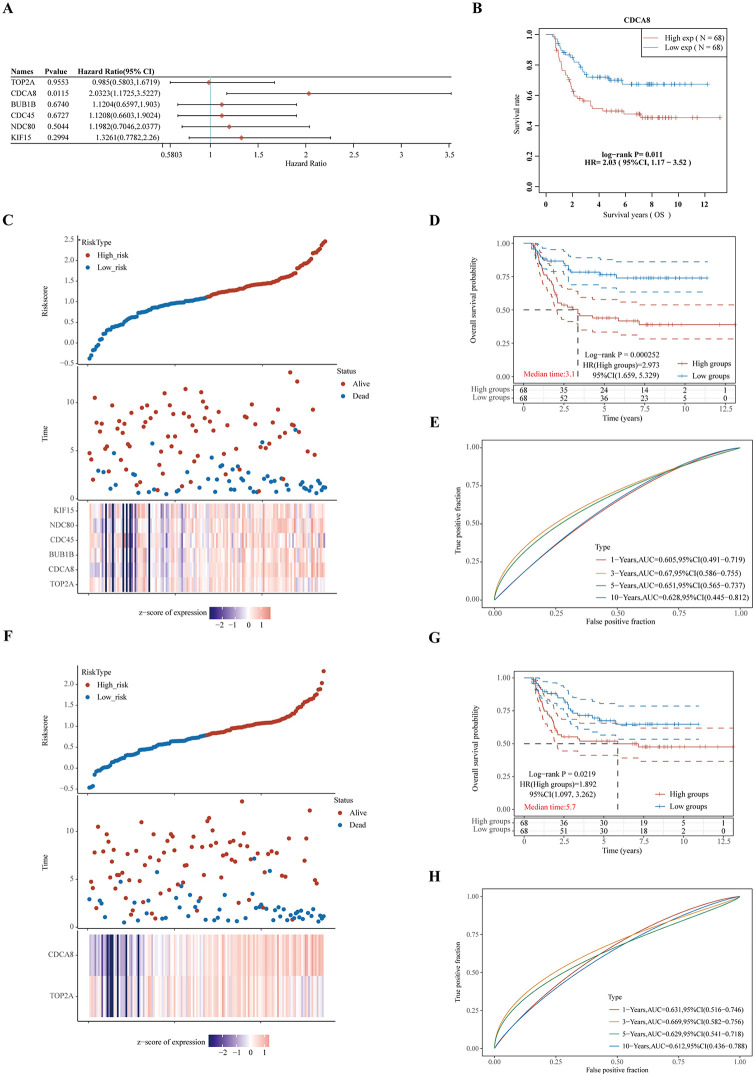
Prognostic analysis of candidate hub genes and construction of risk models in Wilms tumor patients. (A)Univariate Cox regression analysis of the six candidate hub genes for overall survival in WT patients. (B) Kaplan-Meier survival analysis according to CDCA8 expression. Patients were stratified into high and low expression groups using the median CDCA8 expression value. (C-E) Six-gene Cox risk model based on BUB1B, CDC45, CDCA8, KIF15, NDC80, and TOP2A. (C) Risk score distribution, survival status, and gene expression heatmap. (D) Kaplan-Meier survival curves for high- and low-risk groups stratified by the median risk score. (E) Time-dependent ROC curves for the six-gene model at 1, 3, 5, and 10 years. (F-H) Reduced two-gene Cox risk model derived by stepwise regression. (F) Risk score distribution, survival status, and expression heatmap for the reduced model. (G) Kaplan-Meier survival curves for high and low risk groups based on the CDCA8 + TOP2A model. (H) Time-dependent ROC curves for the CDCA8 + TOP2A model at 1, 3, 5, and 10 years.

Univariate Cox regression analysis was performed to evaluate the association between the six candidate hub genes and overall survival in Wilms tumor patients (Fig 5A). Among these genes, CDCA8 was nominally associated with overall survival (p = 0.011, HR = 2.03), with higher CDCA8 expression corresponding to poorer survival outcomes (Fig 5B).

A multivariable Cox regression model was then constructed using the six candidate hub genes to evaluate their combined prognostic relevance (Figs 5C-E). The risk score was calculated as follows: risk score = (−0.5303 × TOP2A expression) + (1.1847 × CDCA8 expression) + (−0.1657 × BUB1B expression) + (−0.2945 × CDC45 expression) + (0.4394 × NDC80 expression) + (−0.2991 × KIF15 expression). Patients were stratified into high-risk and low-risk groups according to the median risk score. The six-gene model separated patients into distinct survival-risk groups, with poorer overall survival observed in the high-risk group (log-rank p = 0.000252, HR = 2.973) (Fig 5D). The median survival time in the high-risk group was 3.1 years. Time-dependent ROC analysis showed AUC values of 0.605, 0.670, 0.651, and 0.628 at 1, 3, 5, and 10 years, respectively (Fig 5E). The AIC value of the six-gene model was 501.8871.

Stepwise Cox regression was subsequently applied to derive a reduced prognostic model (Figs 5F-H). CDCA8 and TOP2A were retained in the final reduced model, with the following risk score formula: risk score = (−0.6449 × TOP2A expression) + (1.0293 × CDCA8 expression). The AIC value of the reduced model was 496.8801. This two-gene model stratified patients into different survival-risk groups in the TARGET-WT cohort, with poorer overall survival observed in the high-risk group (log-rank p = 0.0219, HR = 1.892) (Fig 5G). The median survival time in the high-risk group was 5.7 years. The AUC values of the A model at 1, 3, 5, and 10 years were 0.631, 0.669, 0.629, and 0.612, respectively (Fig 5H). Although the model stratified patients into different survival-risk groups, the time-dependent AUC values ranged from approximately 0.61 to 0.67, indicating modest discriminatory performance. Therefore, this model should be interpreted as exploratory rather than as a validated clinical prognostic tool.

### Comprehensive analysis and external validation of key hub gene expression in wilms tumor patients

**Fig 6:**
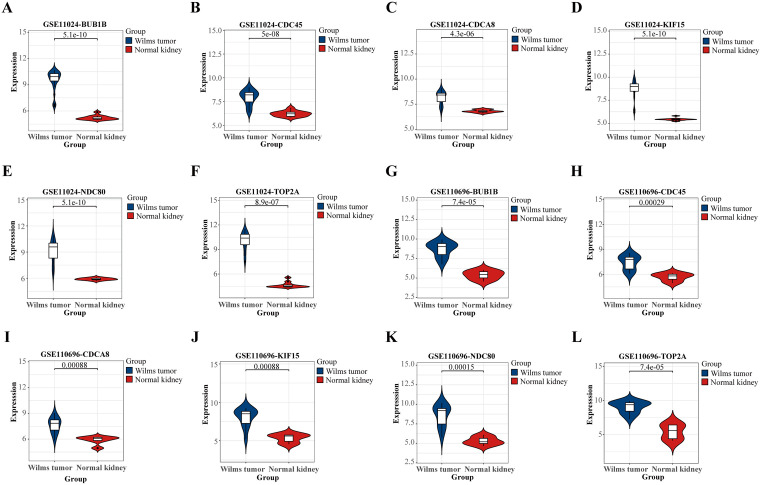
External validation of candidate hub gene expression in WT datasets. (A)-(F) Validation of hub genes performance in external datasets (GSE11024). (G)-(L) Validation of hub genes performance in external datasets (GSE110696).

To further validate the expression patterns of the six candidate hub genes in Wilms tumor, two independent external GEO datasets, GSE11024 and GSE110696, were analyzed. In the GSE11024 cohort, BUB1B, CDC45, CDCA8, KIF15, NDC80, and TOP2A were significantly upregulated in WT tissues compared with normal kidney tissues (Figs 6A-F). Consistent expression patterns were observed in the GSE110696 cohort, in which all six genes also showed increased expression in WT tissues (Figs 6G-L).

These external validation results supported the consistent upregulation of the six candidate hub genes across multiple WT datasets. Together with the TARGET-WT survival analysis and cell-cycle-related enrichment results, CDCA8 was selected for subsequent functional validation.

### Functional validation of CDCA8 in WiT49 cells

**Fig 7:**
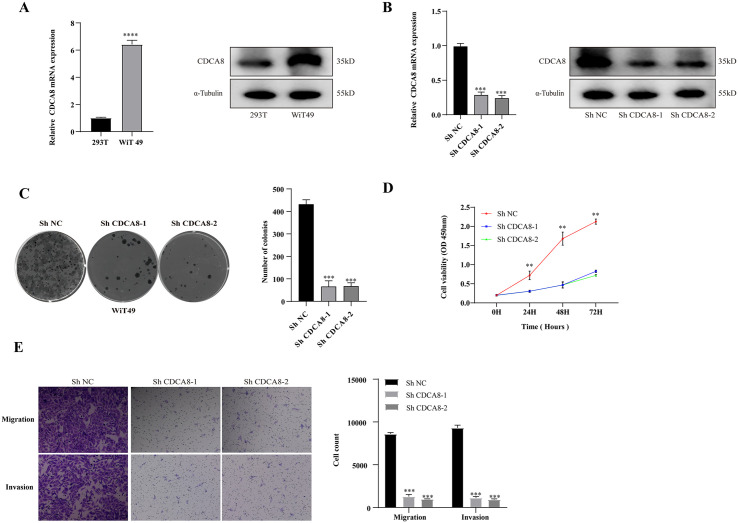
Functional validation of CDCA8 knockdown in WiT49 cells. (A)CDCA8 mRNA and protein expression in WiT49 and HEK 293T cells. (B)CDCA8 knockdown efficiency assessed by RT-qPCR and Western blotting. (C)Colony formation assay in WiT49 cells transfected with shNC or shCDCA8. (D)CCK-8 assay assessing cell viability after CDCA8 knockdown. (E)Transwell migration and invasion assays after CDCA8 knockdown.

CDCA8 expression was first evaluated in WT-related cell models. CDCA8 was found to be upregulated at both the mRNA and protein levels in WiT49 cells compared with HEK 293T cells (Fig 7A). To assess the functional role of CDCA8 in WT cells, a stable lentiviral-mediated CDCA8 knockdown system was established in WiT49 cells. After 72 h of lentiviral infection, CDCA8 knockdown efficiency was confirmed at both the mRNA and protein levels (Fig 7B).

Functional assays showed that CDCA8 knockdown reduced colony formation (Fig 7C) and suppressed cell proliferation (Fig 7D) in WiT49 cells. In addition, transwell assays showed that CDCA8 depletion decreased the migratory and invasive capacities of WiT49 cells (Fig 7E).

These results indicate that CDCA8 knockdown suppresses proliferative, migratory, and invasive phenotypes in WiT49 cells, supporting the selection of CDCA8 for further mechanistic investigation.

### CDCA8 is associated with ATP5F1A in wilms tumor cells

**Fig 8:**
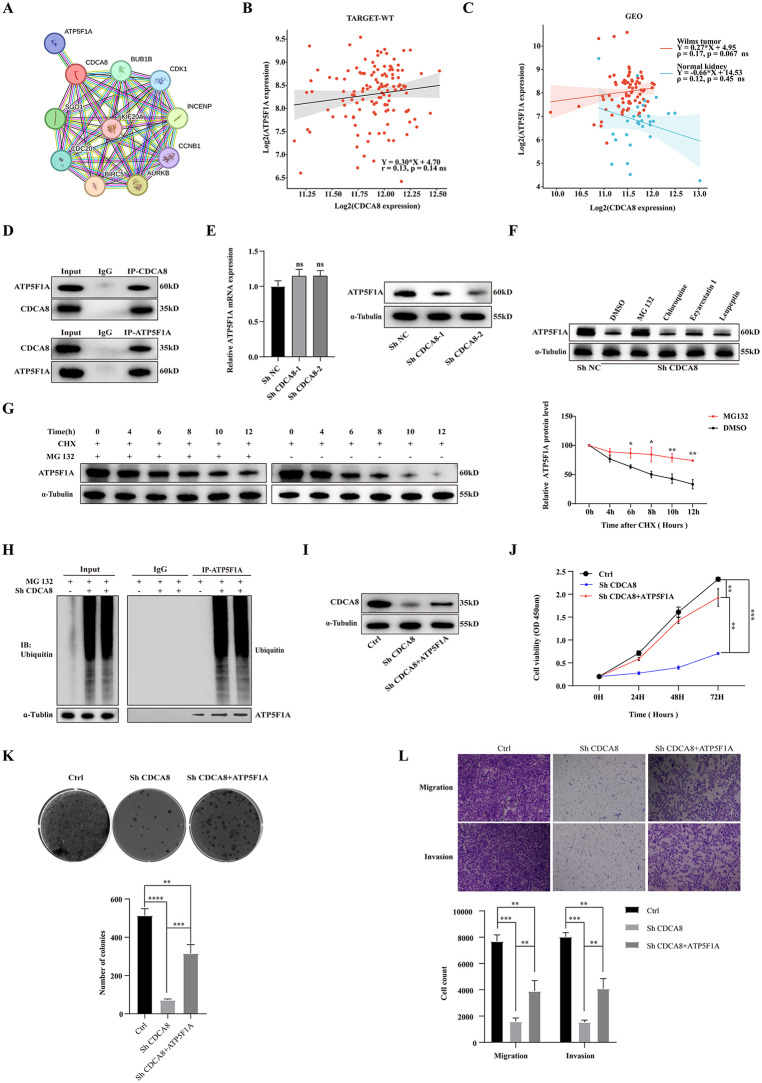
CDCA8 is associated with ATP5F1A and regulates ATP5F1A protein stability in Wilms tumor cells. (A) CDCA8 centered protein-protein interaction (PPI) network generated using the STRING database, showing the top 10 predicted CDCA8 associated proteins. (B) Spearman correlation analysis of CDCA8 and ATP5F1A expression in the TARGET-WT cohort. (C) Spearman correlation analysis of CDCA8 and ATP5F1A expression in the four GEO datasets, stratified by tissue type. (D) Reciprocal co-immunoprecipitation (Co-IP) analysis assessing the protein-level association between CDCA8 and ATP5F1A. (E) ATP5F1A mRNA and protein expression after CDCA8 knockdown. CDCA8 depletion did not markedly alter ATP5F1A mRNA expression but reduced ATP5F1A protein levels. (F) Effects of protein degradation pathway inhibitors on ATP5F1A protein levels in CDCA8-knockdown cells. MG132 treatment partially restored ATP5F1A protein expression after CDCA8 knockdown. (G)Cycloheximide chase assay assessing ATP5F1A protein stability after CDCA8 knockdown, with or without MG132 treatment. (H) Ubiquitination assay of ATP5F1A in control and CDCA8-knockdown cells. ATP5F1A was immunoprecipitated, and ubiquitination levels were detected by Western blotting. Increased ATP5F1A ubiquitination was observed after CDCA8 knockdown under matched MG132-treated conditions. (I) ATP5F1A restoration in CDCA8-knockdown WiT49 cells, with ATP5F1A overexpression confirmed by Western blotting. (J-L) Rescue assays evaluating whether ATP5F1A restoration attenuates the cellular effects induced by CDCA8 knockdown. ATP5F1A restoration partially attenuated the suppression of cell proliferation (J), colony formation (K), and migratory and invasive capacities (L) caused by CDCA8 depletion. Input lanes represent equal amounts of total protein lysate, and normal IgG was used as a negative immunoprecipitation control. For the CHX-chase assay, ATP5F1A band intensities were quantified by densitometry, normalized to α-Tubulin, and expressed relative to the 0 h time point. For the ubiquitination assay, both control and CDCA8-knockdown cells were treated with MG132 under matched conditions before ATP5F1A immunoprecipitation.

To explore potential proteins associated with CDCA8 in Wilms tumor, a CDCA8-centered protein-protein interaction (PPI) network was constructed using the STRING database. The top 10 predicted CDCA8-associated proteins were ATP5F1A, AURKB, BIRC5, BUB1B, CCNB1, CDC20, CDK1, INCENP, KIF20A, and SGO1 (Fig 8A). Among these candidates, ATP5F1A was selected for further analysis because of its potential relevance to mitochondrial energy metabolism and its predicted association with CDCA8.

To evaluate the transcriptomic association between CDCA8 and ATP5F1A, Spearman correlation analyses were performed using the TARGET-WT cohort and the four GEO datasets included in this study. In the TARGET-WT cohort, CDCA8 and ATP5F1A showed a positive co-expression pattern across WT tumor samples (Fig 8B). In the GEO datasets, correlation analyses were further stratified by tissue type. CDCA8 and ATP5F1A showed a positive co-expression pattern in WT tumor tissues, whereas an inverse co-expression pattern was observed in normal kidney tissues (Fig 8C). These transcriptomic associations were not consistently statistically significant across datasets.

Reciprocal co-immunoprecipitation assays were subsequently performed to assess the protein-level association between CDCA8 and ATP5F1A. ATP5F1A was detected in CDCA8 immunoprecipitates, and CDCA8 was detected in ATP5F1A immunoprecipitates (Fig 8D). These findings indicate that CDCA8 and ATP5F1A are present within the same immunoprecipitated protein complex.

### CDCA8 depletion is associated with reduced ATP5F1A protein stability via the ubiquitin-proteasome pathway in wilms tumor cells

To investigate whether CDCA8 regulates ATP5F1A expression, ATP5F1A mRNA and protein levels were examined after CDCA8 knockdown. CDCA8 depletion did not significantly alter ATP5F1A mRNA expression, whereas ATP5F1A protein levels were markedly reduced (Fig 8E), suggesting that CDCA8 may regulate ATP5F1A at the post-transcriptional level.

To determine whether the reduction in ATP5F1A protein was associated with protein degradation pathways, CDCA8-knockdown cells were treated with MG132, chloroquine, Eeyarestatin I, or leupeptin. Among these inhibitors, MG132 restored ATP5F1A protein levels most prominently (Fig 8F), indicating that the decrease in ATP5F1A protein after CDCA8 knockdown was associated, at least in part, with proteasome-mediated degradation. Cycloheximide chase assays were then performed to assess ATP5F1A protein stability. After inhibition of de novo protein synthesis by cycloheximide, ATP5F1A protein abundance decreased more rapidly in CDCA8 knockdown cells than in control cells. MG132 treatment attenuated the reduction of ATP5F1A protein in CDCA8-knockdown cells (Fig 8G), further supporting the involvement of the ubiquitin-proteasome pathway in ATP5F1A protein destabilization following CDCA8 depletion.

To further evaluate ATP5F1A ubiquitination, ATP5F1A was immunoprecipitated from control and CDCA8-knockdown cells, followed by immunoblotting with an anti-ubiquitin antibody. ATP5F1A showed increased ubiquitination in CDCA8-knockdown cells compared with control cells under MG132 treatment (Fig 8H). Together, these results indicate that CDCA8 depletion is associated with increased ATP5F1A ubiquitination and proteasome-associated reduction of ATP5F1A protein, supporting a role for CDCA8 in maintaining ATP5F1A protein stability in Wilms tumor cells.

### ATP5F1A restoration partially attenuates the cellular effects induced by CDCA8 knockdown

Based on the observed association between CDCA8 and ATP5F1A and the effect of CDCA8 knockdown on ATP5F1A protein stability, rescue experiments were performed to determine whether ATP5F1A restoration could attenuate the phenotypic effects induced by CDCA8 depletion. ATP5F1A was overexpressed in CDCA8-knockdown WiT49 cells, and the overexpression efficiency was confirmed by Western blotting (Fig 8I).

Functional assays showed that ATP5F1A overexpression partially restored the cellular phenotypes suppressed by CDCA8 knockdown. Compared with CDCA8-knockdown cells, ATP5F1A overexpression increased cell proliferation (Fig 8J), colony formation (Fig 8K), and migratory and invasive capacities (Fig 8L). These results suggest that ATP5F1A may function as a downstream effector involved in CDCA8 associated regulation of malignant phenotypes in WiT49 cells.

## Discussion

Wilms tumor (WT) is the most common renal malignancy in children, accounting for approximately 5–10% of all pediatric cancers. It is characterized by a complex molecular landscape and highly heterogeneous clinical outcomes [18,19]. Despite significant advances in treatment modalities, including surgery, chemotherapy, and radiation, high-risk WT patients continue to face poor prognosis, primarily due to the challenges of tumor recurrence and metastasis [20–22]. Molecular profiling studies have revealed numerous genetic alterations associated with WT, but the precise molecular mechanisms that drive its progression remain incompletely understood [2,23,24]. Furthermore, the complexity and heterogeneity of WT present a significant challenge in the development of effective treatment strategies.

The identification of candidate biomarkers and molecular mechanisms may help improve understanding of WT heterogeneity and risk stratification [6,25]. However, further validation in independent clinical cohorts and experimental models is required before these findings can be translated into clinical decision-making. In this context, our study aimed to explore candidate prognostic genes and potential functional mechanisms associated with WT progression, with a particular focus on CDCA8, a key regulator of cell cycle progression. Dysregulation of CDCA8 has been implicated in various malignancies, and clarifying its role in WT may provide insight into the contribution of cell-cycle dysregulation to WT biology. Clarifying the role of CDCA8 in WT may help identify candidate molecular mechanisms for further investigation and provide a basis for future validation studies.

In this study, we initially identified six key hub genes—BUB1B, CDC45, CDCA8, KIF15, NDC80, and TOP2A—through comprehensive protein-protein interaction (PPI) network analysis, a widely employed method for identifying critical genes involved in complex diseases. These genes were subsequently validated for their expression in Wilms tumor (WT) tissues using two publicly available datasets (GSE11151 and GSE73209) and the TARGET database, with additional expression validation in GSE11024 and GSE110696. To assess the prognostic relevance of these genes in WT, we performed univariate Cox regression analysis. Our findings revealed that CDCA8, a gene associated with cell-cycle regulation, was nominally associated with overall survival in WT patients. To further evaluate the combined prognostic relevance of these genes, we constructed a risk model using multivariable Cox regression analysis. A stepwise function was applied for iterative model selection, which retained CDCA8 and TOP2A in the reduced model. The reduced model yielded a median survival time of 5.7 years in the high-risk group, with area under the curve (AUC) values of 0.631, 0.669, 0.629, and 0.612 at 1, 3, 5, and 10 years, respectively. These AUC values indicate modest discriminatory performance, and the CDCA8 + TOP2A model should be considered exploratory because it was constructed and evaluated within the TARGET-WT cohort without independent external survival validation. Based on the initial screening, expression validation, and survival analysis, CDCA8 was selected for subsequent functional investigation. Although BUB1B was also upregulated in DAWT compared with FHWT, CDCA8 was prioritized because of its survival association and limited prior characterization in WT. The biological role of BUB1B in WT remains worthy of further study.

Cell division cycle-associated protein 8 (CDCA8) is a crucial component of the chromosome passenger complex (CPC) and a key regulator of mitosis and cytokinesis [26–28]. Dysregulation of CDCA8 has been implicated in the progression of various cancers, including hepatocellular carcinoma [11,29], bladder cancer [9], prostate cancer [10], pancreatic cancer [30], and gliomas [31]. However, its role in Wilms tumor (WT), particularly in promoting malignant progression, remains uninvestigated. Previous studies have shown that CDCA8 knockdown inhibits hepatocellular carcinoma (HCC) cell proliferation, colony formation, and migration through suppression of the MEK/ERK pathway [29]. In bladder cancer, CDCA8 stabilizes HIF1α by competing with PTEN for binding to AKT, which displaces PTEN and activates the AKT/GSK3β signaling axis, thereby enhancing HIF1α stability and promoting cancer cell survival [9]. Consistent with the known role of CDCA8 in cell-cycle regulation, our functional experiments showed that CDCA8 knockdown suppressed proliferation, colony formation, migration, and invasion in WiT49 cells, supporting a functional role for CDCA8 in WT cell phenotypes.

ATP5F1A encodes a subunit of the mitochondrial ATP synthase, which catalyzes ATP synthesis during oxidative phosphorylation (OXPHOS) by utilizing the electrochemical gradient of protons across the inner mitochondrial membrane [12]. Tumor cells often rely on OXPHOS, adapting mitochondrial bioenergetic pathways to optimize ATP production, thereby fueling various metabolic processes essential for cancer cell survival and proliferation [32,33]. While ATP synthase typically generates ATP by utilizing the transmembrane proton gradient, this reaction can be reversed under acidic or hypoxic conditions, leading to ATP hydrolysis. Previous studies have shown that the oncogenic kinase TNK2/ACK1 phosphorylates ATP5F1A at Tyr243/246, selectively enhancing prostate cancer cell survival while inducing mitochondrial vulnerability [34]. Furthermore, the chimeric protein SFT2D2-TBX19, which encodes TBX19–202, stabilizes mitochondrial ATP synthase via ATP5F1A phosphorylation, promoting prostate cancer progression [35]. In the present study, PPI-based screening identified ATP5F1A as a candidate CDCA8 associated molecule. Spearman correlation analyses showed a positive co-expression pattern between CDCA8 and ATP5F1A in WT tumor samples, whereas an inverse co-expression pattern was observed in normal kidney tissues. At the protein level, reciprocal Co-IP assays demonstrated that CDCA8 and ATP5F1A were present within the same immunoprecipitated protein complex, supporting a protein-level association between these two molecules. Further studies will be needed to define the precise structural basis of this association and to determine whether additional intermediary proteins are involved.

Stem cell-like characteristics in Wilms tumor (WT) suggest that changes in the renal developmental transcriptome may serve as critical drivers of WT initiation and progression, with differences in cellular differentiation and energy metabolism distinguishing WT from normal renal development [36]. Additionally, the high expression of inhibin in WT is closely associated with chemotherapy resistance, as it modulates apoptotic factors in the mitochondria, affecting the apoptosis of cancer cells [37]. High-resolution rotating nuclear magnetic resonance spectroscopy has revealed significant alterations in glycolysis, glutamine metabolism, the TCA cycle, and lipid and branched-chain amino acid metabolism in WT [38]. Gene ontology (GO) and KEGG pathway enrichment analyses of differentially expressed genes (DEGs) in WT demonstrated that these genes are closely involved in small molecule catabolism, carboxylic acid metabolism, and transmembrane transport. Further KEGG pathway analysis highlighted the enrichment of pathways related to DNA replication, the cell cycle, and base excision repair. Together with the functional data, these findings suggest a possible connection between cell-cycle dysregulation and mitochondrial metabolic regulation in WT cells.

In summary, this study developed an exploratory prognostic risk model based on six candidate hub genes using public transcriptomic and clinical data. The prognostic model stratified patients into different survival-risk groups in the TARGET-WT cohort, but its time-dependent AUC values indicated only modest discriminatory performance. CDCA8 was identified as a candidate prognostic and functional regulator in WT. By analyzing clinical data from public databases and performing cellular functional experiments, we further explored the role of CDCA8 in WT and found that CDCA8 knockdown suppressed malignant phenotypes in WiT49 cells. The current data suggest that CDCA8 helps maintain ATP5F1A protein stability. CDCA8 depletion was associated with increased ATP5F1A ubiquitination and proteasome-associated reduction of ATP5F1A protein. ATP5F1A restoration partially attenuated the inhibitory effects of CDCA8 knockdown on WiT49 cell proliferation, colony formation, migration, and invasion. Several limitations should be considered. First, although multiple public WT cohorts were included, the analyses were retrospective and were constrained by the limited availability of datasets with matched transcriptomic, histological, survival, and treatment information. The discovery datasets also contained relatively small normal kidney comparator groups, which may affect the stability of DEG identification. Second, the prognostic models were developed mainly using the TARGET-WT cohort. Although external GEO datasets supported the expression patterns of the candidate hub genes, independent survival validation of the reduced CDCA8 + TOP2A model remains limited by the availability of comparable WT cohorts. Third, functional validation was performed primarily in WiT49 cells because experimentally tractable WT models are limited. Further validation in additional WT models, patient-derived specimens, and in vivo systems would help strengthen the biological relevance of CDCA8. Finally, the present data support a role for CDCA8 in maintaining ATP5F1A protein stability, but the upstream ubiquitination machinery and the precise structural basis of the CDCA8-ATP5F1A association remain to be further characterized.

## Conclusions

This study developed an exploratory prognostic risk model based on six candidate hub genes using public transcriptomic and clinical data. CDCA8 was identified as a candidate prognostic and functional regulator in WT. Functional experiments in WiT49 cells showed that CDCA8 knockdown suppressed malignant cellular phenotypes. The current data suggest that CDCA8 helps maintain ATP5F1A protein stability. CDCA8 depletion was associated with increased ATP5F1A ubiquitination and proteasome-associated reduction of ATP5F1A protein. ATP5F1A restoration partially attenuated the inhibitory effects of CDCA8 knockdown on WiT49 cell proliferation, colony formation, migration, and invasion. These findings support ATP5F1A as a candidate downstream effector of CDCA8 and provide preliminary evidence for a CDCA8–ATP5F1A regulatory axis in WT.

## Supporting information

S1 File S1 Table. The detailed list of proteins included in the PPI network. S2 Table. List of hub proteins ranked by Degree, MCC, and MNC algorithms and proteins included in the top-ranked MCODE module of the PPI network.
(ZIP)

S2 File Original Images for BlotsGels. Original uncropped and unadjusted blot images underlying the Western blot and immunoprecipitation results presented in the manuscript. The file includes the raw images corresponding to the blot-based figures used to support the reported CDCA8, ATP5F1A, ubiquitination, and loading-control results.
(PDF)
