# Supplementary material for: CDCA8 regulates ATP5F1A protein stability and malignant phenotypes in wilms tumor cells: Prognostic implications and mechanistic insights
Source: PLoS One. 2026 Jul 21;21(7):e0353696. doi: 10.1371/journal.pone.0353696 (PMC13387503; doi:10.1371/journal.pone.0353696)
Supplement: S1 File — S2 Table. List of hub proteins ranked by Degree, MCC, and MNC algorithms and proteins included in the top-ranked MCODE module of the PPI network. (ZIP) [file pone.0353696.s001.zip › Sup Table2 List of hub proteins ranked by Degree, MCC, and MNC algorithms and proteins included in the top-ranked MCODE module of the PPI network.docx]

**Sup Table 2. List of hub proteins ranked by Degree, MCC, and MNC algorithms and proteins included in the top-ranked MCODE module of the PPI network.**

| **Top 20 in network string_interactions_short.tsv ranked by Degree method** |  |  | **Top 20 in network string_interactions_short.tsv ranked by MNC method** |  |  | **Top 20 in network string_interactions_short.tsv ranked by MCC method** |  |  | **MCODE Cluster1-score 33.2** |
| --- | --- | --- | --- | --- | --- | --- | --- | --- | --- |
| **Rank** | **Name** | **Score** | **Rank** | **Name** | **Score** | **Rank** | **Name** | **Score** | STIL |
| 1 | CDH1 | 79 | 1 | CDH1 | 74 | 1 | TOP2A | 1.94E+26 | CENPW |
| 2 | SDHA | 69 | 2 | SDHA | 69 | 1 | CENPF | 1.94E+26 | KIF14 |
| 3 | TOP2A | 68 | 3 | TOP2A | 66 | 3 | BUB1B | 1.94E+26 | MCM4 |
| 4 | EZH2 | 64 | 4 | EZH2 | 62 | 3 | KIF15 | 1.94E+26 | CDCA8 |
| 5 | HIF1A | 60 | 5 | CDCA8 | 60 | 5 | CDCA8 | 1.94E+26 | TPX2 |
| 5 | SUCLG1 | 60 | 6 | SUCLG1 | 59 | 5 | NUSAP1 | 1.94E+26 | DSCC1 |
| 5 | CDCA8 | 60 | 7 | HIF1A | 57 | 7 | PBK | 1.94E+26 | SKA3 |
| 8 | ACO2 | 57 | 7 | ACO2 | 57 | 8 | ASPM | 1.94E+26 | CDKN3 |
| 8 | IDH2 | 57 | 9 | IDH2 | 56 | 9 | MELK | 1.94E+26 | HMMR |
| 8 | BUB1B | 57 | 9 | BUB1B | 56 | 10 | TPX2 | 1.94E+26 | NUSAP1 |
| 11 | EGF | 55 | 11 | HADHB | 52 | 11 | RAD51AP1 | 1.94E+26 | MELK |
| 12 | HADHB | 52 | 12 | RFC4 | 50 | 12 | CDCA5 | 1.94E+26 | TROAP |
| 12 | CDC45 | 52 | 12 | CDC45 | 50 | 13 | CDC45 | 1.94E+26 | GINS2 |
| 14 | RFC4 | 51 | 14 | MCM4 | 49 | 14 | UBE2T | 1.94E+26 | FANCI |
| 15 | MCM4 | 49 | 15 | ALDH4A1 | 47 | 15 | NDC80 | 1.94E+26 | UBE2T |
| 15 | HMMR | 49 | 16 | EGF | 46 | 16 | HMMR | 1.94E+26 | TOP2A |
| 17 | NDC80 | 48 | 16 | NDC80 | 46 | 17 | CEP55 | 1.94E+26 | RAD51AP1 |
| 17 | ACADM | 48 | 16 | FANCI | 46 | 18 | PTTG1 | 1.94E+26 | MCM2 |
| 19 | ALDH4A1 | 47 | 16 | KIF15 | 46 | 19 | CDKN3 | 1.94E+26 | MND1 |
| 19 | KIF15 | 47 | 16 | CENPF | 46 | 20 | DTL | 1.87E+26 | CENPU |
|  |  |  |  |  |  |  |  |  | CEP55 |
|  |  |  |  |  |  |  |  |  | PBK |
|  |  |  |  |  |  |  |  |  | ASPM |
|  |  |  |  |  |  |  |  |  | RFC4 |
|  |  |  |  |  |  |  |  |  | PRC1 |
|  |  |  |  |  |  |  |  |  | HJURP |
|  |  |  |  |  |  |  |  |  | NDC80 |
|  |  |  |  |  |  |  |  |  | CENPF |
|  |  |  |  |  |  |  |  |  | BUB1B |
|  |  |  |  |  |  |  |  |  | CKAP2L |
|  |  |  |  |  |  |  |  |  | PTTG1 |
|  |  |  |  |  |  |  |  |  | DTL |
|  |  |  |  |  |  |  |  |  | CDC45 |
|  |  |  |  |  |  |  |  |  | KIF15 |
|  |  |  |  |  |  |  |  |  | CDCA5 |
