# Supplementary material for: CDCA8 regulates ATP5F1A protein stability and malignant phenotypes in wilms tumor cells: Prognostic implications and mechanistic insights
Source: PLoS One. 2026 Jul 21;21(7):e0353696. doi: 10.1371/journal.pone.0353696 (PMC13387503; doi:10.1371/journal.pone.0353696)
Supplement: S1 File — S2 Table. List of hub proteins ranked by Degree, MCC, and MNC algorithms and proteins included in the top-ranked MCODE module of the PPI network. (ZIP) [file pone.0353696.s001.zip › Sup Table1 The detailed list of proteins included in the PPI network.docx]

**Sup Table 1. The detailed list of proteins included in the PPI network.**

| **ALL-Common elements in GSE11151 GSE73209 :** | **UP-Common elements in GSE11151 GSE73209 :** | **DOWN-Common elements in GSE11151 GSE73209 :** |
| --- | --- | --- |
| CEMIP | CEMIP | TMEM52B |
| SIX1 | SIX1 | GAL3ST1 |
| TIAM1 | TIAM1 | CLDN8 |
| OSR1 | OSR1 | MAL |
| SIX2 | SIX2 | CDH16 |
| PHLDA1 | PHLDA1 | ERP27 |
| CELSR3 | CELSR3 | TFCP2L1 |
| CRABP2 | CRABP2 | ELF5 |
| SIX4 | SIX4 | RASGRP1 |
| ELOVL2 | ELOVL2 | CA10 |
| FAIM2 | FAIM2 | MME |
| ENAH | ENAH | RAB25 |
| VWCE | VWCE | NPHS2 |
| ADGRG2 | ADGRG2 | C1ORF116 |
| CHN1 | CHN1 | ITGA2 |
| SHMT2 | SHMT2 | CLDN19 |
| FAM216A | FAM216A | SMIM24 |
| PCDHB10 | PCDHB10 | EMCN |
| AP1S2 | AP1S2 | HNF1B |
| THBS4 | THBS4 | CCDC198 |
| CBX5 | CBX5 | FOXQ1 |
| RNF217 | RNF217 | CALB1 |
| SMARCC1 | SMARCC1 | TMEM139 |
| CALHM6 | CALHM6 | MECOM |
| PCDH19 | PCDH19 | GATA3 |
| ENC1 | ENC1 | ATP6V0A4 |
| CKAP4 | CKAP4 | FXYD2 |
| SNRPB | SNRPB | MUC1 |
| IGF2 | IGF2 | COL4A4 |
| ROR2 | ROR2 | KCNJ1 |
| TBC1D31 | TBC1D31 | PTPRO |
| GPAM | BOC | IRX1 |
| BOC | NPTX2 | KCNJ16 |
| NPTX2 | LAMA4 | TMEM45B |
| LAMA4 | CHAF1B | TMEM213 |
| CHAF1B | PMAIP1 | PTH1R |
| PMAIP1 | CTHRC1 | SCIN |
| CTHRC1 | DESI2 | IRX2 |
| DESI2 | DSCC1 | DPP4 |
| DSCC1 | MEOX1 | CGNL1 |
| MEOX1 | EYA1 | LRP2 |
| EYA1 | SNRPF | VTCN1 |
| SNRPF | ZNF426 | CRYBG1 |
| ZNF426 | HES6 | TMEM207 |
| HES6 | NME1 | PRR15L |
| NME1 | ZMAT4 | F2RL1 |
| ZMAT4 | CENPW | CGN |
| CENPW | OLFML2B | PLA2R1 |
| OLFML2B | FAT3 | COBLL1 |
| FAT3 | MCM2 | GLS |
| MCM2 | APOC1 | TRPM4 |
| APOC1 | SEPTIN6 | NTN4 |
| SEPTIN6 | PCSK5 | TMEM125 |
| PCSK5 | EGFLAM | GALNT14 |
| EGFLAM | TENM3 | USH1C |
| TENM3 | TIMELESS | NEURL3 |
| TIMELESS | VASH2 | RIPOR1 |
| VASH2 | LRRN1 | RERGL |
| MYH1 | KAT6A | AOC1 |
| LRRN1 | SOX11 | KCNJ15 |
| KAT6A | RGS17 | TFAP2B |
| SOX11 | NAA16 | PLEKHH2 |
| RGS17 | RNASEH2A | PODXL |
| NAA16 | MYO9B | FAM3B |
| RNASEH2A | SV2A | PAQR5 |
| MYO9B | CCND2 | TMPRSS2 |
| SV2A | MCM4 | IRX5 |
| CCND2 | TEX10 | KL |
| MCM4 | ZNF521 | NPNT |
| TEX10 | PRIM1 | VAV3 |
| ZNF521 | MDK | ESRRG |
| PRIM1 | RCOR2 | FAM149A |
| MDK | TROAP | ASPA |
| RCOR2 | SEM1 | SCNN1B |
| TROAP | MAP3K12 | C21ORF62 |
| SEM1 | AGO2 | CA4 |
| MAP3K12 | PRAME | TNFRSF11B |
| AGO2 | ASPM | GPR160 |
| PRAME | SCG5 | GRB14 |
| ASPM | PRDX4 | ESAM |
| SCG5 | RPS21 | ANXA4 |
| PRDX4 | SACS | SMTNL2 |
| RPS21 | GLIPR2 | AP1M2 |
| SACS | RGS4 | MST1 |
| GLIPR2 | POLB | COL4A3 |
| RGS4 | STIL | PPP1R16B |
| POLB | LRP4 | PLEKHA6 |
| STIL | MND1 | CWH43 |
| LRP4 | NUSAP1 | DEFB1 |
| MND1 | CCDC34 | ATP1A1 |
| NUSAP1 | NDC80 | NDRG1 |
| CCDC34 | DYRK2 | DDN |
| NDC80 | CDCA8 | EVPL |
| DYRK2 | PDPN | ACSM3 |
| CDCA8 | RBFOX2 | DAB2 |
| PDPN | GAS1 | HPGD |
| RBFOX2 | CHTF18 | SLC48A1 |
| GAS1 | SPC24 | MARVELD3 |
| CHTF18 | HMCN1 | LRATD2 |
| SPC24 | MSANTD3 | CA2 |
| HMCN1 | SGCD | SLC44A3 |
| MSANTD3 | SKA3 | ARHGAP24 |
| SGCD | PLXNA3 | CDH1 |
| SKA3 | NINJ2 | SOST |
| PLXNA3 | FBL | PRODH |
| NINJ2 | SKP2 | GK |
| FBL | TPX2 | CLCNKB |
| SKP2 | SSBP4 | CTSH |
| TPX2 | ZNF423 | CLDN10 |
| SSBP4 | UBE2T | MUC20 |
| ZNF423 | LYAR | CLDN16 |
| UBE2T | MELK | HNF4G |
| LYAR | DNMT1 | UGT8 |
| MELK | GPC2 | NOSTRIN |
| DNMT1 | CDKN3 | OSBPL3 |
| GPC2 | EID3 | CLRN3 |
| CDKN3 | C1ORF112 | TFAP2A |
| EID3 | PPP1R18 | TM7SF2 |
| C1ORF112 | DDX39A | VEPH1 |
| PPP1R18 | CACNB3 | HBB |
| DDX39A | TACC3 | PTPRB |
| CACNB3 | CEP78 | SULT1C2 |
| TACC3 | NUP107 | CRYL1 |
| CEP78 | SNRPD1 | RAPGEF3 |
| NUP107 | PTGFRN | CRYZ |
| SNRPD1 | PTTG1 | SCNN1G |
| PTGFRN | RADIL | REN |
| PTTG1 | KDM1A | PLLP |
| RADIL | NUDT1 | LRRC19 |
| KDM1A | COLGALT2 | GABRA2 |
| NUDT1 | DTL | ELOVL7 |
| COLGALT2 | IRF2BP2 | BSPRY |
| DTL | RAD51AP1 | P3H2 |
| IRF2BP2 | PLA2G7 | WDR72 |
| RAD51AP1 | DKC1 | HOGA1 |
| PLA2G7 | TRANK1 | GPX3 |
| DKC1 | ARGLU1 | GSTM3 |
| TRANK1 | THOC6 | ALDH1A1 |
| ARGLU1 | SAP30 | PPL |
| THOC6 | PLEKHO1 | ECHDC2 |
| SAP30 | TCF4 | COX7A1 |
| PLEKHO1 | HJURP | SLC18B1 |
| TCF4 | CDCA5 | IRX3 |
| HJURP | EZH2 | ANXA9 |
| CDCA5 | PRPF19 | RBM47 |
| EZH2 | NELL2 | ILDR1 |
| PRPF19 | HSF2 | LGALS2 |
| NELL2 | RFC4 | MAN1A1 |
| HSF2 | HK2 | UMOD |
| RFC4 | CENPL | SLCO4C1 |
| HK2 | DDX55 | COQ9 |
| CENPL | CEP55 | SPX |
| DDX55 | NRBP1 | DNER |
| CEP55 | COP1 | ENPEP |
| NRBP1 | DTNA | KBTBD11 |
| COP1 | TMEM108 | AGTR1 |
| DTNA | HRK | CRHBP |
| TMEM108 | PRC1 | TSPAN12 |
| HRK | TPP2 | TMC4 |
| PRC1 | PRRC2C | ASS1 |
| TPP2 | DTX3 | TSPAN1 |
| PRRC2C | GLIPR1 | MAP7 |
| DTX3 | SRP19 | ADGRF1 |
| GLIPR1 | CDH2 | EMX1 |
| SRP19 | VRK1 | LMBRD1 |
| CDH2 | MAP4K4 | SLC16A5 |
| VRK1 | HNRNPAB | CPEB4 |
| MAP4K4 | TIMP1 | MYO9A |
| HNRNPAB | INTS8 | SLC4A4 |
| TIMP1 | FANCI | GPHN |
| INTS8 | YBX3 | PLS1 |
| FANCI | C1ORF21 | AKTIP |
| YBX3 | NAP1L1 | HIBCH |
| C1ORF21 | CDK2AP1 | FGL2 |
| NAP1L1 | MN1 | RNF144B |
| CDK2AP1 | LIG1 | PPARGC1A |
| MN1 | AEN | ACE2 |
| LIG1 | TBX15 | FMO1 |
| AEN | PTPN12 | KIF12 |
| TBX15 | USP14 | GULP1 |
| PTPN12 | KCTD3 | PTPN3 |
| USP14 | MEX3B | TMEM37 |
| KCTD3 | TLCD5 | TINAG |
| MEX3B | PRR3 | AIF1L |
| TLCD5 | TUBA1A | SCNN1A |
| PRR3 | TOP2A | STK32B |
| TUBA1A | CDC45 | CA12 |
| TOP2A | POLE3 | A4GALT |
| CDC45 | CCDC88A | KIF13B |
| POLE3 | CTPS1 | GATA2 |
| CCDC88A | DOCK9 | GLDC |
| CTPS1 | TRIAP1 | SLC16A4 |
| DOCK9 | POLD1 | WDR91 |
| TRIAP1 | GTF2E2 | MPP5 |
| POLD1 | CENPF | AQP2 |
| GTF2E2 | REL | DDC |
| CENPF | HEATR6 | SLC7A7 |
| REL | PBK | PLCL1 |
| HEATR6 | NXT1 | TSPAN7 |
| PBK | NEMP1 | ATP1B1 |
| NXT1 | ILF3 | FECH |
| NEMP1 | CCDC59 | GLRX |
| ILF3 | GPR180 | TENT5C |
| CCDC59 | AVEN | CAVIN2 |
| GPR180 | NKX3-2 | NDUFS7 |
| AVEN | DUSP12 | SLC3A1 |
| NKX3-2 | P3H3 | PDZK1 |
| DUSP12 | HENMT1 | MAF |
| P3H3 | ENO2 | TEK |
| ZNF576 | HSPA14 | BOK |
| HENMT1 | GINS2 | CUBN |
| ENO2 | FRMD6 | LYPD6B |
| HSPA14 | PPP1CC | UGT3A1 |
| GINS2 | STXBP5 | ADAP1 |
| FRAT2 | ANKDD1A | SPINK1 |
| FRMD6 | SPATS2 | RAB11FIP5 |
| PPP1CC | PMS1 | FGFR3 |
| STXBP5 | KPNA3 | BBOX1 |
| ANKDD1A | MCUB | CDS1 |
| SPATS2 | RBP1 | CASR |
| PMS1 | HNRNPR | CYP4V2 |
| KPNA3 | POGLUT2 | TBX2 |
| MCUB | OGFRL1 | KCNJ10 |
| RBP1 | ASNS | CX3CL1 |
| HNRNPR | P3H1 | UGT2A3 |
| POGLUT2 | BUB1B | APCDD1L |
| OGFRL1 | SH3PXD2A | HOXB7 |
| ASNS | PRRX2 | FRK |
| P3H1 | HNRNPLL | CMTM4 |
| BUB1B | PRKAB2 | TSPAN8 |
| FADD | NARF | ZNF385B |
| SH3PXD2A | SMC3 | CLDN2 |
| PRRX2 | RPL14 | IMPA2 |
| HNRNPLL | NABP2 | ANK3 |
| PRKAB2 | CKAP2L | VIL1 |
| NARF | NOP2 | NQO1 |
| SMC3 | CAPRIN1 | SLC13A1 |
| RPL14 | CENPU | COBL |
| NABP2 | FSCN1 | CHCHD10 |
| CKAP2L | RBM3 | CYFIP2 |
| NOP2 | ZBTB33 | KLHL3 |
| CAPRIN1 | GRAMD1A | LAD1 |
| CENPU | CILP | KIAA1522 |
| FSCN1 | BCL11A | C4ORF19 |
| RBM3 | ENTPD4 | GCA |
| ZBTB33 | TOPBP1 | OGDHL |
| GRAMD1A | HMMR | ST3GAL6 |
| CILP | PODN | TMEM174 |
| BCL11A | KDM5A | TMEM30B |
| ENTPD4 | POLQ | TM4SF18 |
| TOPBP1 | CBX1 | ATP6V1B1 |
| HMMR | FAM53C | CAPN3 |
| PODN | GFPT2 | CLIC5 |
| KDM5A | PPP1R12A | BHMT |
| POLQ | BTF3L4 | ENPP4 |
| CBX1 | TCERG1 | ARHGEF16 |
| FAM53C | KIF14 | ADGRG1 |
| GFPT2 | DNAJC2 | EPHX2 |
| PPP1R12A | KPNA2 | EMX2 |
| BTF3L4 | LRFN5 | MPC2 |
| TCERG1 | IL13RA1 | PHYHD1 |
| KIF14 | CHD1 | GLUD1 |
| DNAJC2 | SLC25A14 | SLC44A4 |
| KPNA2 | ETV6 | AGXT2 |
| LRFN5 | STAT4 | SLC27A2 |
| IL13RA1 | CPXM1 | TPD52L1 |
| CHD1 | MARCKS | SDHA |
| SLC25A14 | NOP56 | MSRB1 |
| ETV6 | RPF2 | ASB9 |
| STAT4 | DROSHA | HKDC1 |
| CPXM1 | RSL1D1 | IQGAP2 |
| MARCKS | MTF2 | SHANK2 |
| NOP56 | UFC1 | HPN |
| PLIN1 | RBM34 | ARSD |
| RPF2 | COLGALT1 | SLC4A9 |
| DROSHA | KIF15 | SLC4A1 |
| RSL1D1 | CRY1 | IVNS1ABP |
| MTF2 | RGS1 | ITM2B |
| UFC1 | PHF21A | ATP6V1D |
| RBM34 |  | ATP6V1A |
| COLGALT1 |  | LZTS3 |
| KIF15 |  | ASRGL1 |
| CRY1 |  | PTGER3 |
| RGS1 |  | GGT6 |
| PHF21A |  | RAB11FIP3 |
| TMEM52B |  | ACAD8 |
| GAL3ST1 |  | SLC25A1 |
| CLDN8 |  | SLC37A4 |
| MAL |  | FRMD3 |
| CDH16 |  | WWC1 |
| ERP27 |  | SLC17A1 |
| TFCP2L1 |  | PVALB |
| ELF5 |  | RAB40B |
| RASGRP1 |  | ACMSD |
| CA10 |  | FCAMR |
| MME |  | C11ORF54 |
| RAB25 |  | ALDH4A1 |
| NPHS2 |  | FGF9 |
| C1ORF116 |  | ACADM |
| ITGA2 |  | XPNPEP2 |
| CLDN19 |  | GOT1 |
| SMIM24 |  | GPC5 |
| EMCN |  | PHYHIPL |
| HNF1B |  | ECRG4 |
| CCDC198 |  | BCAR1 |
| FOXQ1 |  | CLDN7 |
| CALB1 |  | SH3BGRL2 |
| TMEM139 |  | SUMF1 |
| MECOM |  | NAPRT |
| GATA3 |  | WNK4 |
| ATP6V0A4 |  | NEDD4L |
| FXYD2 |  | ACO2 |
| MUC1 |  | CISH |
| COL4A4 |  | CAPS |
| KCNJ1 |  | CDKL2 |
| PTPRO |  | ACY3 |
| IRX1 |  | PDZK1IP1 |
| KCNJ16 |  | SLC34A1 |
| TMEM45B |  | COX4I1 |
| TMEM213 |  | EPCAM |
| PTH1R |  | SLC12A3 |
| SCIN |  | AUH |
| IRX2 |  | ELMO3 |
| DPP4 |  | JUP |
| CGNL1 |  | SEMA4D |
| LRP2 |  | CLUH |
| VTCN1 |  | PBLD |
| CRYBG1 |  | FBP1 |
| TMEM207 |  | CNDP2 |
| PRR15L |  | SLC44A2 |
| F2RL1 |  | EZR |
| CGN |  | SHANK3 |
| PLA2R1 |  | ACAD11 |
| COBLL1 |  | HSD17B8 |
| GLS |  | HNMT |
| TRPM4 |  | GALM |
| NTN4 |  | CAT |
| TMEM125 |  | PALM |
| GALNT14 |  | PRSS8 |
| USH1C |  | SLC22A5 |
| NEURL3 |  | TACSTD2 |
| RIPOR1 |  | COX7B |
| RERGL |  | SDC4 |
| AOC1 |  | STX3 |
| KCNJ15 |  | ENAM |
| TFAP2B |  | TCN2 |
| PLEKHH2 |  | SLC43A2 |
| PODXL |  | COMTD1 |
| FAM3B |  | ZC2HC1C |
| PAQR5 |  | ATP5IF1 |
| TMPRSS2 |  | MPPED2 |
| IRX5 |  | RAB17 |
| KL |  | UPP2 |
| NPNT |  | FOLR3 |
| VAV3 |  | DDAH1 |
| ESRRG |  | BDH2 |
| FAM149A |  | SLC22A11 |
| ASPA |  | PRKCQ |
| SCNN1B |  | SLC23A1 |
| C21ORF62 |  | CLDN11 |
| CA4 |  | KMO |
| TNFRSF11B |  | ABCD3 |
| GPR160 |  | RALYL |
| GRB14 |  | FAM107B |
| ESAM |  | FOLR1 |
| ANXA4 |  | SLC1A1 |
| SMTNL2 |  | SOWAHA |
| AP1M2 |  | GALNT11 |
| MST1 |  | CHDH |
| COL4A3 |  | SAT2 |
| PPP1R16B |  | SEMA3B |
| PLEKHA6 |  | AQP1 |
| CWH43 |  | HOXB8 |
| DEFB1 |  | GHITM |
| ATP1A1 |  | CRB3 |
| NDRG1 |  | TOX3 |
| DDN |  | ZDHHC6 |
| EVPL |  | CYB5D2 |
| ACSM3 |  | SLC22A8 |
| DAB2 |  | SOSTDC1 |
| HPGD |  | HAO2 |
| SLC48A1 |  | SLC7A9 |
| MARVELD3 |  | WLS |
| LRATD2 |  | ADHFE1 |
| CA2 |  | SLC22A4 |
| SLC44A3 |  | ABCB1 |
| ARHGAP24 |  | ABHD14B |
| CDH1 |  | ZDHHC14 |
| SOST |  | HSPA2 |
| PRODH |  | TSPAN33 |
| GK |  | PRSS35 |
| CLCNKB |  | ADIRF |
| CTSH |  | FAM83H |
| CLDN10 |  | DAZAP2 |
| MUC20 |  | MFSD4A |
| CLDN16 |  | PRRG1 |
| HNF4G |  | CRIM1 |
| UGT8 |  | PNP |
| NOSTRIN |  | GRAMD1C |
| OSBPL3 |  | STX7 |
| CLRN3 |  | HSDL2 |
| TFAP2A |  | MCCC1 |
| TM7SF2 |  | CPNE8 |
| VEPH1 |  | HDHD3 |
| HBB |  | TOM1L1 |
| PTPRB |  | SLC5A1 |
| SULT1C2 |  | TCTA |
| CRYL1 |  | MUC15 |
| RAPGEF3 |  | CRYM |
| CRYZ |  | SLC17A3 |
| SCNN1G |  | DEPTOR |
| REN |  | CCDC115 |
| PLLP |  | ACAT1 |
| LRRC19 |  | ACSS1 |
| GABRA2 |  | SERPINI1 |
| ELOVL7 |  | GGT1 |
| BSPRY |  | EHD3 |
| P3H2 |  | MAOB |
| WDR72 |  | SLC39A11 |
| HOGA1 |  | SUCNR1 |
| GPX3 |  | OMA1 |
| GSTM3 |  | TMEM141 |
| ALDH1A1 |  | KNG1 |
| PPL |  | GLYAT |
| ECHDC2 |  | LDHB |
| COX7A1 |  | VAMP8 |
| SLC18B1 |  | DECR1 |
| IRX3 |  | SPTLC2 |
| ANXA9 |  | PANK1 |
| RBM47 |  | ATP5PD |
| ILDR1 |  | SLC6A13 |
| LGALS2 |  | GIPC2 |
| MAN1A1 |  | SFXN2 |
| UMOD |  | PRKAB1 |
| SLCO4C1 |  | TMEM38B |
| COQ9 |  | NUP62CL |
| SPX |  | COMMD8 |
| DNER |  | AP5M1 |
| ENPEP |  | WBP2 |
| KBTBD11 |  | CYB5A |
| AGTR1 |  | LDHD |
| CRHBP |  | SLC6A12 |
| TSPAN12 |  | SYT13 |
| TMC4 |  | SLC16A9 |
| ASS1 |  | AIFM1 |
| TSPAN1 |  | SUCLG1 |
| MAP7 |  | SPR |
| ADGRF1 |  | PRODH2 |
| EMX1 |  | SLC39A5 |
| LMBRD1 |  | ABHD17C |
| SLC16A5 |  | THEM6 |
| CPEB4 |  | ATP6V1G3 |
| MYO9A |  | TPRG1L |
| SLC4A4 |  | GPAT3 |
| GPHN |  | SLC5A11 |
| PLS1 |  | ERMP1 |
| AKTIP |  | PTGR1 |
| HIBCH |  | DPYS |
| FGL2 |  | ETFDH |
| RNF144B |  | CAPN12 |
| PPARGC1A |  | DNASE1 |
| ACE2 |  | ACSF2 |
| FMO1 |  | GLYCTK |
| KIF12 |  | GPD1 |
| GULP1 |  | MGLL |
| PTPN3 |  | SH3GL2 |
| TMEM37 |  | ATP12A |
| TINAG |  | PCCA |
| AIF1L |  | CYSTM1 |
| SCNN1A |  | CIDEB |
| STK32B |  | BLVRB |
| CA12 |  | MAST4 |
| A4GALT |  | PDZD3 |
| KIF13B |  | PRTN3 |
| GATA2 |  | MTFR1L |
| GLDC |  | SLC35F5 |
| SLC16A4 |  | HMGCL |
| WDR91 |  | DMTN |
| MPP5 |  | MBP |
| AQP2 |  | ATP6V1H |
| DDC |  | SLC30A2 |
| SLC7A7 |  | RNF186 |
| PLCL1 |  | DUSP9 |
| TSPAN7 |  | MT1F |
| ATP1B1 |  | ETFB |
| FECH |  | MAOA |
| GLRX |  | NR1H4 |
| TENT5C |  | HSD17B14 |
| CAVIN2 |  | NAPEPLD |
| NDUFS7 |  | PLCH2 |
| SLC3A1 |  | SLC22A6 |
| PDZK1 |  | SLC51B |
| MAF |  | PRRG2 |
| TEK |  | ALDH8A1 |
| BOK |  | CNPPD1 |
| CUBN |  | PPP1R1A |
| LYPD6B |  | PC |
| UGT3A1 |  | APLNR |
| ADAP1 |  | MYO6 |
| SPINK1 |  | FCGRT |
| RAB11FIP5 |  | NDRG2 |
| FGFR3 |  | HS6ST2 |
| BBOX1 |  | NINL |
| CDS1 |  | BCO1 |
| CASR |  | ATP5F1C |
| CYP4V2 |  | ENPP5 |
| TBX2 |  | SUOX |
| KCNJ10 |  | ATP6AP2 |
| CX3CL1 |  | APOM |
| UGT2A3 |  | ECH1 |
| APCDD1L |  | ALDH1L1 |
| HOXB7 |  | TMEM88 |
| FRK |  | NQO2 |
| CMTM4 |  | GFM1 |
| TSPAN8 |  | TNFRSF21 |
| ZNF385B |  | SMIM1 |
| CLDN2 |  | ZMYND12 |
| IMPA2 |  | AMT |
| ANK3 |  | IDNK |
| VIL1 |  | CADPS2 |
| NQO1 |  | LRRC75B |
| SLC13A1 |  | SLC13A3 |
| COBL |  | STRADB |
| CHCHD10 |  | EFNA1 |
| CYFIP2 |  | IDH2 |
| KLHL3 |  | AMACR |
| LAD1 |  | MTURN |
| KIAA1522 |  | HOOK2 |
| C4ORF19 |  | FABP3 |
| GCA |  | SERPINF2 |
| OGDHL |  | TGOLN2 |
| ST3GAL6 |  | CYP17A1 |
| TMEM174 |  | DHCR24 |
| TMEM30B |  | RALB |
| TM4SF18 |  | C12ORF75 |
| ATP6V1B1 |  | PEPD |
| CAPN3 |  | FTH1 |
| CLIC5 |  | NDUFA4 |
| BHMT |  | RAB29 |
| ENPP4 |  | STARD8 |
| ARHGEF16 |  | EGF |
| ADGRG1 |  | PAQR7 |
| EPHX2 |  | AIG1 |
| EMX2 |  | L2HGDH |
| MPC2 |  | MCRIP2 |
| PHYHD1 |  | CLTRN |
| GLUD1 |  | HMGCS2 |
| SLC44A4 |  | ALPL |
| AGXT2 |  | KHK |
| SLC27A2 |  | ASB13 |
| TPD52L1 |  | NAPSA |
| SDHA |  | PRR13 |
| MSRB1 |  | SLC5A10 |
| ASB9 |  | ENPP6 |
| HKDC1 |  | ENTPD5 |
| IQGAP2 |  | ERBB3 |
| SHANK2 |  | TMBIM6 |
| HPN |  | TRAPPC4 |
| ARSD |  | PROZ |
| SLC4A9 |  | DPEP1 |
| SLC4A1 |  | FLOT2 |
| IVNS1ABP |  | ECI2 |
| ITM2B |  | BPHL |
| ATP6V1D |  | SEPHS2 |
| ATP6V1A |  | PKP4 |
| LZTS3 |  | RENBP |
| ASRGL1 |  | SLC26A4 |
| PTGER3 |  | EFHD1 |
| GGT6 |  | NUCB1 |
| RAB11FIP3 |  | MRGPRF |
| ANXA1 |  | SLC22A2 |
| ACAD8 |  | SOX18 |
| SLC25A1 |  | ATP6V0A1 |
| SLC37A4 |  | NEU1 |
| FRMD3 |  | AP2A2 |
| WWC1 |  | SDHD |
| SLC17A1 |  | CTDSPL |
| PVALB |  | KIAA1191 |
| RAB40B |  | SLC25A5 |
| ACMSD |  | SLC10A2 |
| FCAMR |  | AGMAT |
| C11ORF54 |  | RTN4 |
| ALDH4A1 |  | IL17RB |
| FGF9 |  | RABL3 |
| ACADM |  | NPR3 |
| XPNPEP2 |  | SLC47A2 |
| GOT1 |  | FRAS1 |
| GPC5 |  | SLC45A3 |
| PHYHIPL |  | ZFP3 |
| ECRG4 |  | CARD10 |
| BCAR1 |  | CTXN3 |
| CLDN7 |  | SERPINA5 |
| SH3BGRL2 |  | FXYD4 |
| SUMF1 |  | GATM |
| NAPRT |  | ATP6V0D1 |
| WNK4 |  | ACADS |
| NEDD4L |  | KCNK5 |
| ACO2 |  | COX20 |
| CISH |  | PIGV |
| CAPS |  | HADH |
| CDKL2 |  | TLN2 |
| ACY3 |  | CEACAM1 |
| PDZK1IP1 |  | HSD11B2 |
| SLC34A1 |  | ACOX2 |
| COX4I1 |  | SUSD2 |
| EPCAM |  | ENDOG |
| SLC12A3 |  | FAM151A |
| AUH |  | DMGDH |
| ELMO3 |  | SKAP1 |
| JUP |  | UQCRC1 |
| SEMA4D |  | PRPS2 |
| CLUH |  | GSTO2 |
| PBLD |  | CASTOR1 |
| FBP1 |  | LRRC28 |
| CNDP2 |  | SLC5A2 |
| SLC44A2 |  | HIBADH |
| EZR |  | GJB2 |
| SHANK3 |  | MAL2 |
| ACAD11 |  | TPCN2 |
| HSD17B8 |  | PRKCA |
| HNMT |  | ACADL |
| GALM |  | IQCK |
| CAT |  | LYG1 |
| PALM |  | ENOSF1 |
| PRSS8 |  | CYB561D2 |
| SLC22A5 |  | CYP4A11 |
| TACSTD2 |  | SLC16A10 |
| COX7B |  | KIF3B |
| SDC4 |  | RCAN1 |
| STX3 |  | OPLAH |
| ENAM |  | DHDH |
| TCN2 |  | HADHB |
| SLC43A2 |  | BHMT2 |
| COMTD1 |  | CXCL14 |
| ZC2HC1C |  | ALDOB |
| ATP5IF1 |  | NOX4 |
| MPPED2 |  | ZDHHC23 |
| RAB17 |  | MIOX |
| UPP2 |  | MT1H |
| FOLR3 |  | ADH6 |
| DDAH1 |  | HOXB6 |
| BDH2 |  | DUSP3 |
| SLC22A11 |  | METTL7B |
| PRKCQ |  | TRIM6 |
| SLC23A1 |  | LYPLAL1 |
| CLDN11 |  | CFLAR |
| KMO |  | TMEM150A |
| ABCD3 |  | RDH11 |
| RALYL |  | TMBIM1 |
| FAM107B |  | ALDH6A1 |
| FOLR1 |  | A1CF |
| SLC1A1 |  | ZNF684 |
| SOWAHA |  | STARD10 |
| GALNT11 |  | CMBL |
| CHDH |  | SUSD3 |
| SAT2 |  | SLC22A18 |
| SEMA3B |  | ATP5F1B |
| AQP1 |  | PAQR8 |
| HOXB8 |  | MSMO1 |
| GHITM |  | ATP5PB |
| CRB3 |  | OSBP |
| TOX3 |  | TRPM6 |
| ZDHHC6 |  | PCBD1 |
| CYB5D2 |  | MOCS2 |
| SLC22A8 |  | COASY |
| SOSTDC1 |  | DHTKD1 |
| HAO2 |  | ABHD14A |
| SLC7A9 |  | DENND2D |
| WLS |  | PLCG2 |
| ADHFE1 |  | NABP1 |
| SLC22A4 |  | CLN5 |
| ABCB1 |  | SLC9A3R1 |
| ABHD14B |  | LHX1 |
| ZDHHC14 |  | PPFIBP2 |
| HSPA2 |  | MTHFS |
| TSPAN33 |  | SLC7A13 |
| PRSS35 |  | AQP7 |
| ADIRF |  | SUGCT |
| FAM83H |  | TBC1D13 |
| DAZAP2 |  | ETFRF1 |
| MFSD4A |  | GSTK1 |
| PRRG1 |  | RIDA |
| CRIM1 |  | ATP6V0E2 |
| PNP |  | SLC6A18 |
| GRAMD1C |  | EVA1A |
| STX7 |  | NTNG1 |
| HSDL2 |  | ZHX3 |
| MCCC1 |  | CD9 |
| CPNE8 |  | FMO4 |
| HDHD3 |  | OCEL1 |
| TOM1L1 |  | PNPO |
| SLC5A1 |  |  |
| TCTA |  |  |
| MUC15 |  |  |
| CRYM |  |  |
| SLC17A3 |  |  |
| DEPTOR |  |  |
| CCDC115 |  |  |
| ACAT1 |  |  |
| ACSS1 |  |  |
| SERPINI1 |  |  |
| GGT1 |  |  |
| EHD3 |  |  |
| MAOB |  |  |
| SLC39A11 |  |  |
| SUCNR1 |  |  |
| OMA1 |  |  |
| TMEM141 |  |  |
| KNG1 |  |  |
| GLYAT |  |  |
| LDHB |  |  |
| VAMP8 |  |  |
| DECR1 |  |  |
| SPTLC2 |  |  |
| PANK1 |  |  |
| ATP5PD |  |  |
| SLC6A13 |  |  |
| GIPC2 |  |  |
| SFXN2 |  |  |
| PRKAB1 |  |  |
| TMEM38B |  |  |
| NUP62CL |  |  |
| COMMD8 |  |  |
| DDX50 |  |  |
| AP5M1 |  |  |
| WBP2 |  |  |
| CYB5A |  |  |
| LDHD |  |  |
| SLC6A12 |  |  |
| SYT13 |  |  |
| SLC16A9 |  |  |
| AIFM1 |  |  |
| CD47 |  |  |
| SUCLG1 |  |  |
| SPR |  |  |
| PRODH2 |  |  |
| SLC39A5 |  |  |
| ABHD17C |  |  |
| THEM6 |  |  |
| ATP6V1G3 |  |  |
| TPRG1L |  |  |
| GPAT3 |  |  |
| SLC5A11 |  |  |
| ERMP1 |  |  |
| PTGR1 |  |  |
| DPYS |  |  |
| ETFDH |  |  |
| CAPN12 |  |  |
| DNASE1 |  |  |
| ACSF2 |  |  |
| GLYCTK |  |  |
| GPD1 |  |  |
| MGLL |  |  |
| SH3GL2 |  |  |
| ATP12A |  |  |
| PCCA |  |  |
| CYSTM1 |  |  |
| CIDEB |  |  |
| BLVRB |  |  |
| MAST4 |  |  |
| PDZD3 |  |  |
| PRTN3 |  |  |
| MTFR1L |  |  |
| SLC35F5 |  |  |
| HMGCL |  |  |
| DMTN |  |  |
| MBP |  |  |
| ATP6V1H |  |  |
| SLC30A2 |  |  |
| RNF186 |  |  |
| DUSP9 |  |  |
| MT1F |  |  |
| ETFB |  |  |
| MAOA |  |  |
| NR1H4 |  |  |
| HSD17B14 |  |  |
| NAPEPLD |  |  |
| PLCH2 |  |  |
| SLC22A6 |  |  |
| SLC51B |  |  |
| PRRG2 |  |  |
| ALDH8A1 |  |  |
| CNPPD1 |  |  |
| PPP1R1A |  |  |
| PC |  |  |
| APLNR |  |  |
| MYO6 |  |  |
| FCGRT |  |  |
| NDRG2 |  |  |
| HS6ST2 |  |  |
| NINL |  |  |
| BCO1 |  |  |
| ATP5F1C |  |  |
| ENPP5 |  |  |
| SUOX |  |  |
| ATP6AP2 |  |  |
| APOM |  |  |
| ECH1 |  |  |
| KTN1 |  |  |
| ALDH1L1 |  |  |
| TMEM88 |  |  |
| NQO2 |  |  |
| PTGR2 |  |  |
| GFM1 |  |  |
| TNFRSF21 |  |  |
| SMIM1 |  |  |
| ZMYND12 |  |  |
| AMT |  |  |
| IDNK |  |  |
| CADPS2 |  |  |
| LRRC75B |  |  |
| SLC13A3 |  |  |
| STRADB |  |  |
| EFNA1 |  |  |
| IDH2 |  |  |
| AMACR |  |  |
| MTURN |  |  |
| HOOK2 |  |  |
| FABP3 |  |  |
| SERPINF2 |  |  |
| TGOLN2 |  |  |
| CYP17A1 |  |  |
| DHCR24 |  |  |
| RALB |  |  |
| C12ORF75 |  |  |
| PEPD |  |  |
| FTH1 |  |  |
| NDUFA4 |  |  |
| RAB29 |  |  |
| STARD8 |  |  |
| EGF |  |  |
| PAQR7 |  |  |
| AIG1 |  |  |
| L2HGDH |  |  |
| MCRIP2 |  |  |
| CLTRN |  |  |
| HMGCS2 |  |  |
| ALPL |  |  |
| KHK |  |  |
| ASB13 |  |  |
| NAPSA |  |  |
| PRR13 |  |  |
| SLC5A10 |  |  |
| ENPP6 |  |  |
| ENTPD5 |  |  |
| ERBB3 |  |  |
| TMBIM6 |  |  |
| TRAPPC4 |  |  |
| PROZ |  |  |
| DPEP1 |  |  |
| FLOT2 |  |  |
| ECI2 |  |  |
| BPHL |  |  |
| SEPHS2 |  |  |
| PKP4 |  |  |
| RENBP |  |  |
| SLC26A4 |  |  |
| EFHD1 |  |  |
| NUCB1 |  |  |
| MRGPRF |  |  |
| NUCB2 |  |  |
| SLC22A2 |  |  |
| SOX18 |  |  |
| ATP6V0A1 |  |  |
| NEU1 |  |  |
| AP2A2 |  |  |
| SDHD |  |  |
| CTDSPL |  |  |
| KIAA1191 |  |  |
| SLC25A5 |  |  |
| SLC10A2 |  |  |
| AGMAT |  |  |
| RTN4 |  |  |
| IL17RB |  |  |
| RABL3 |  |  |
| NPR3 |  |  |
| SLC47A2 |  |  |
| FRAS1 |  |  |
| SLC45A3 |  |  |
| ZFP3 |  |  |
| CARD10 |  |  |
| CTXN3 |  |  |
| SERPINA5 |  |  |
| FXYD4 |  |  |
| GATM |  |  |
| ATP6V0D1 |  |  |
| ACADS |  |  |
| KCNK5 |  |  |
| COX20 |  |  |
| PIGV |  |  |
| HADH |  |  |
| TLN2 |  |  |
| CEACAM1 |  |  |
| HSD11B2 |  |  |
| ACOX2 |  |  |
| SUSD2 |  |  |
| ENDOG |  |  |
| FAM151A |  |  |
| DMGDH |  |  |
| SKAP1 |  |  |
| UQCRC1 |  |  |
| PRPS2 |  |  |
| GSTO2 |  |  |
| CASTOR1 |  |  |
| LRRC28 |  |  |
| SLC5A2 |  |  |
| HIBADH |  |  |
| GJB2 |  |  |
| MAL2 |  |  |
| TPCN2 |  |  |
| PRKCA |  |  |
| ACADL |  |  |
| IQCK |  |  |
| LYG1 |  |  |
| ENOSF1 |  |  |
| CYB561D2 |  |  |
| CYP4A11 |  |  |
| SLC16A10 |  |  |
| KIF3B |  |  |
| RCAN1 |  |  |
| OPLAH |  |  |
| NPIPB15 |  |  |
| DHDH |  |  |
| HADHB |  |  |
| BHMT2 |  |  |
| KLF4 |  |  |
| CXCL14 |  |  |
| ALDOB |  |  |
| NOX4 |  |  |
| ZDHHC23 |  |  |
| MIOX |  |  |
| MT1H |  |  |
| ADH6 |  |  |
| HOXB6 |  |  |
| DUSP3 |  |  |
| METTL7B |  |  |
| TRIM6 |  |  |
| LYPLAL1 |  |  |
| CFLAR |  |  |
| TMEM150A |  |  |
| RDH11 |  |  |
| TMBIM1 |  |  |
| ALDH6A1 |  |  |
| A1CF |  |  |
| ZNF684 |  |  |
| STARD10 |  |  |
| CMBL |  |  |
| SUSD3 |  |  |
| SLC22A18 |  |  |
| ATP5F1B |  |  |
| PAQR8 |  |  |
| MSMO1 |  |  |
| ATP5PB |  |  |
| AEBP1 |  |  |
| OSBP |  |  |
| HIF1A |  |  |
| TRPM6 |  |  |
| PCBD1 |  |  |
| MOCS2 |  |  |
| COASY |  |  |
| DHTKD1 |  |  |
| ABHD14A |  |  |
| DENND2D |  |  |
| PLCG2 |  |  |
| NABP1 |  |  |
| CLN5 |  |  |
| SLC9A3R1 |  |  |
| LHX1 |  |  |
| PPFIBP2 |  |  |
| MTHFS |  |  |
| SLC7A13 |  |  |
| AQP7 |  |  |
| SUGCT |  |  |
| TBC1D13 |  |  |
| NMD3 |  |  |
| ETFRF1 |  |  |
| GSTK1 |  |  |
| RIDA |  |  |
| ATP6V0E2 |  |  |
| SLC6A18 |  |  |
| EVA1A |  |  |
| NTNG1 |  |  |
| ZHX3 |  |  |
| CD9 |  |  |
| FMO4 |  |  |
| OCEL1 |  |  |
| PNPO |  |  |
