# Supplementary material for: CDCA8 regulates ATP5F1A protein stability and malignant phenotypes in wilms tumor cells: Prognostic implications and mechanistic insights
Source: PLoS One. 2026 Jul 21;21(7):e0353696. doi: 10.1371/journal.pone.0353696 (PMC13387503; doi:10.1371/journal.pone.0353696)
Supplement: S2 File — Original uncropped and unadjusted blot images underlying the Western blot and immunoprecipitation results presented in the manuscript. The file includes the raw images corresponding to the blot-based figures used to support the reported CDCA8, ATP5F1A, ubiquitination, and loading-control results. (PDF) [file pone.0353696.s002.pdf]

Figure 7-A: Functional validation of CDCA8 in WiT-49 cells-Main.

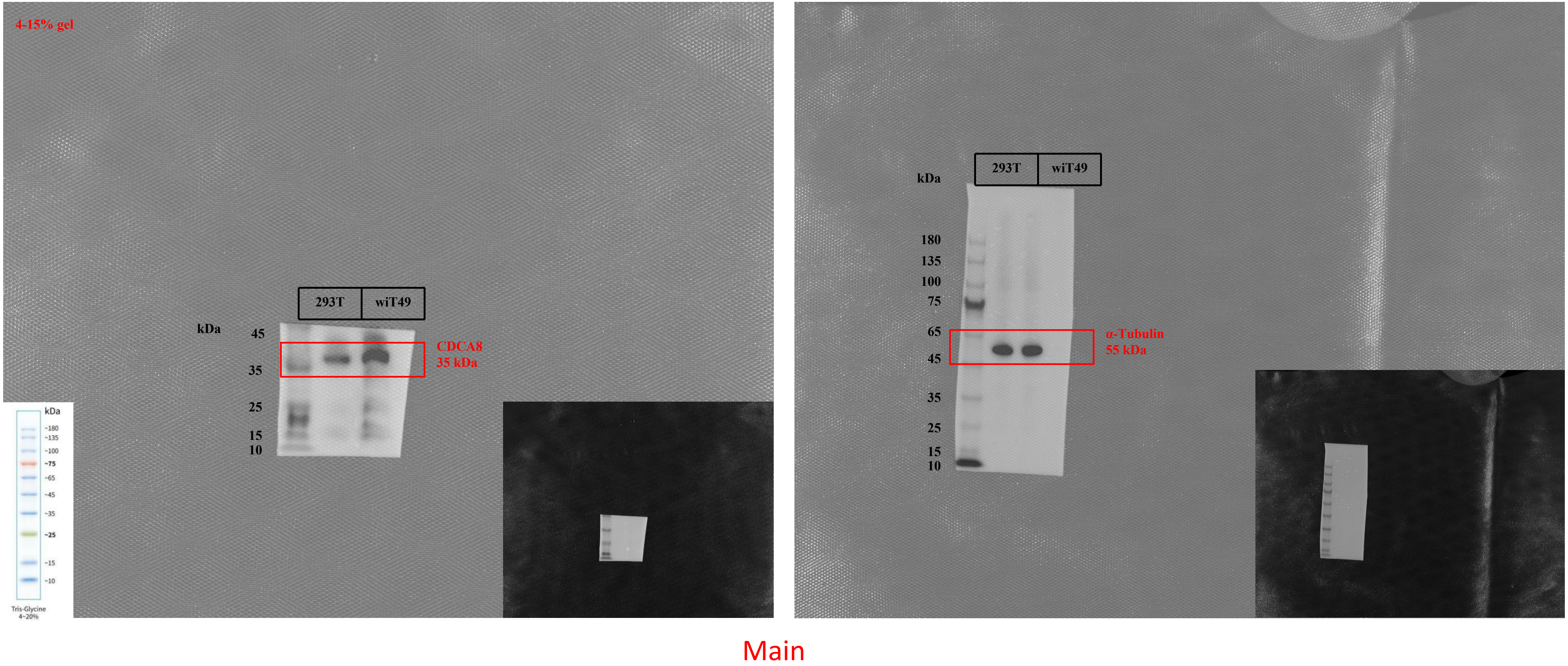

(A)Expression analysis of the CDCA8 gene in the cell line.

Figure 7-A: Functional validation of CDCA8 in WiT-49 cells-Repeat.

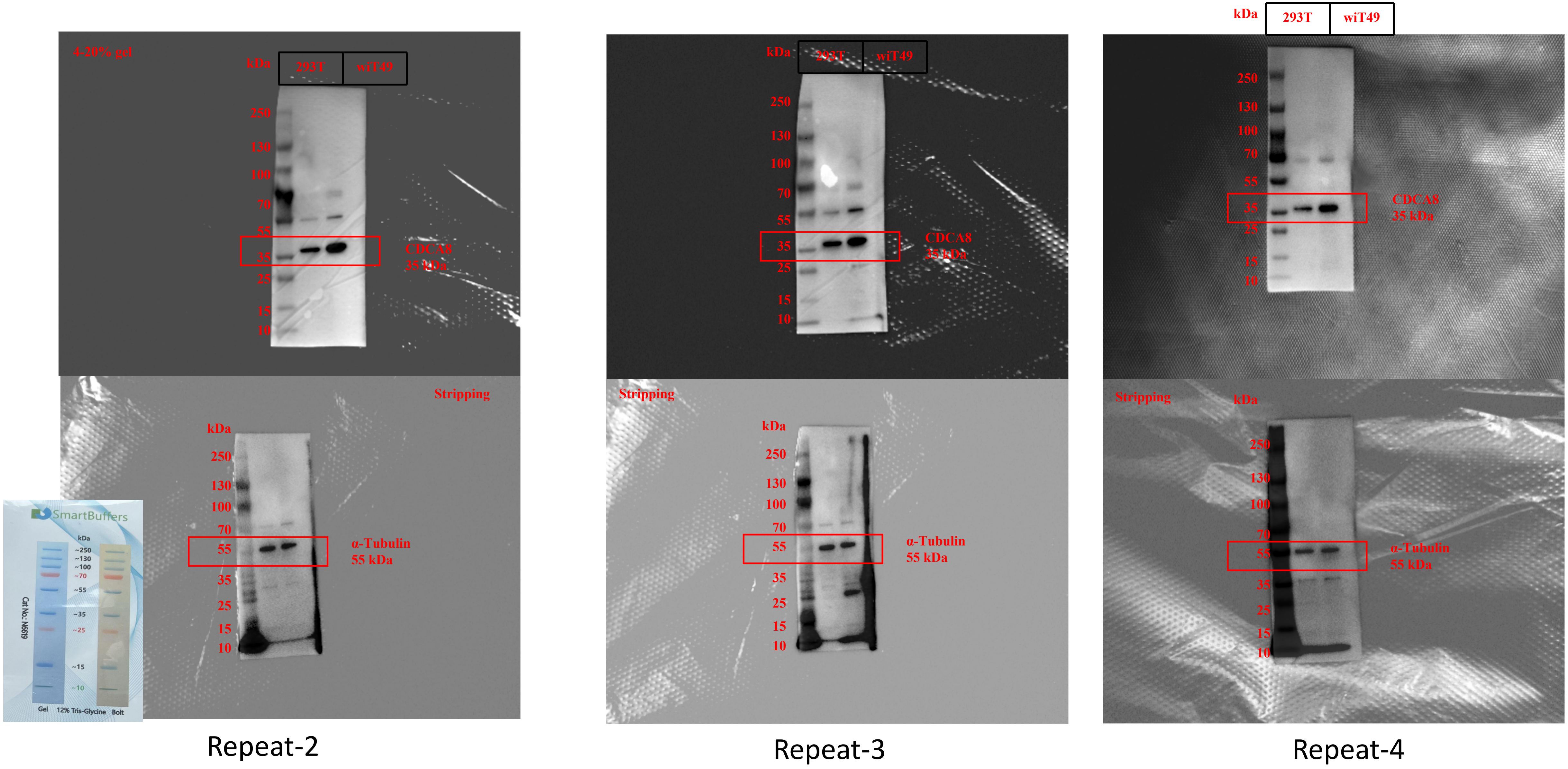

(A)Expression analysis of the CDCA8 gene in the cell line.

Figure 7-B: Functional validation of CDCA8 in WiT-49 cells-Main.

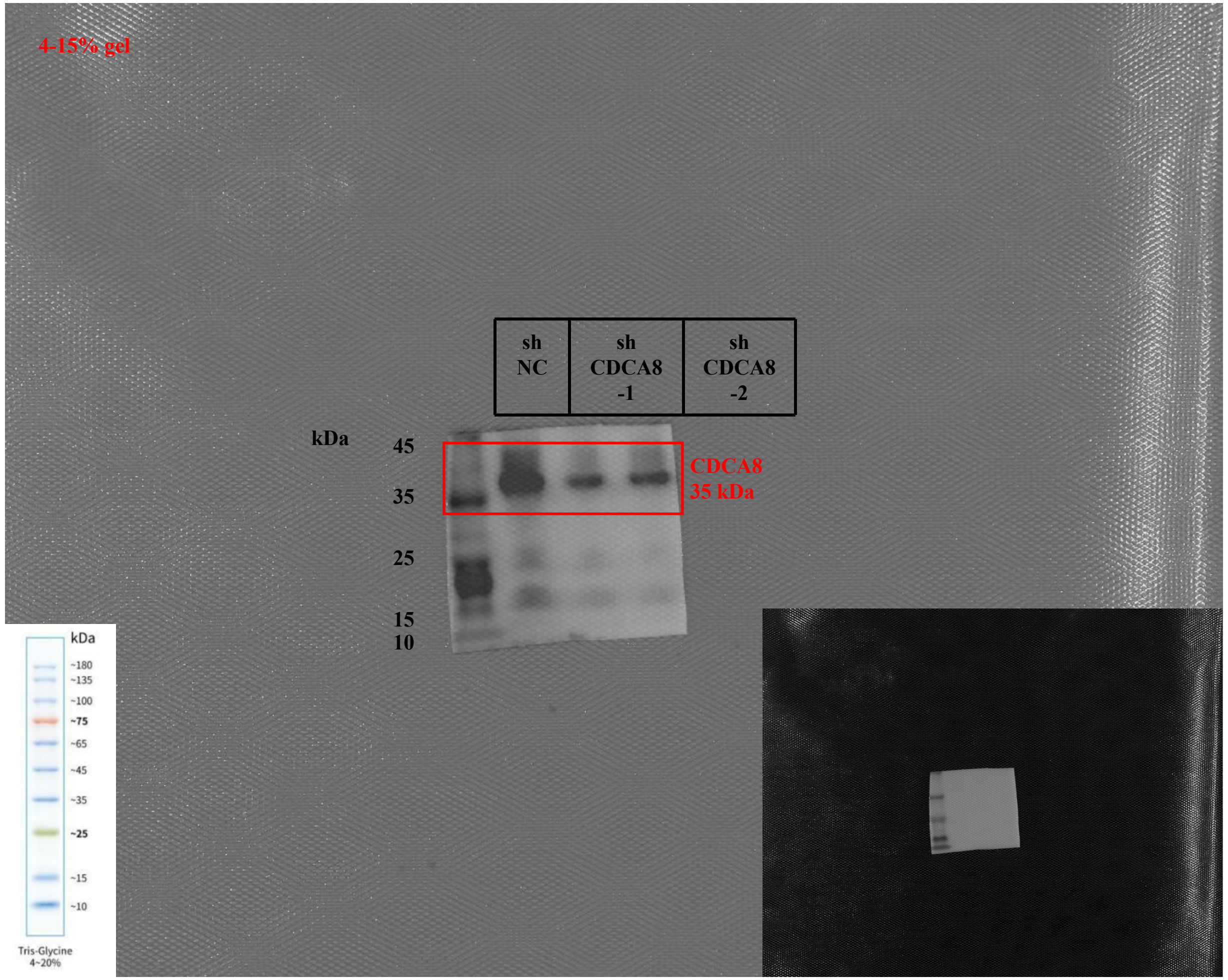

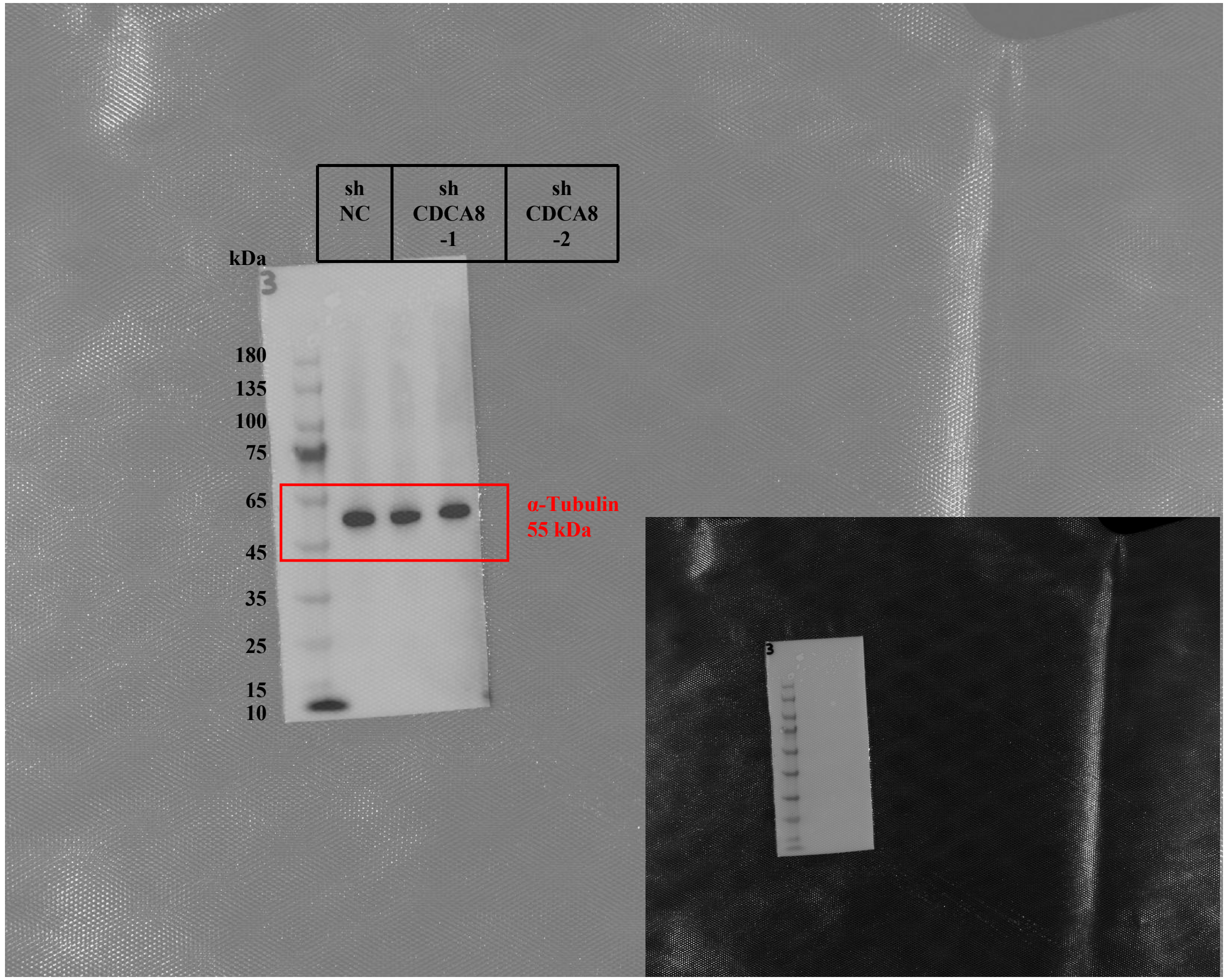

Main

(B)WiT-49 cells were transfected with CDCA8 sh RNA or sh NC. Relative protein expression of CDCA8 was detected by Western blot.

Figure 7-B: Functional validation of CDCA8 in WiT-49 cells-Repeat.

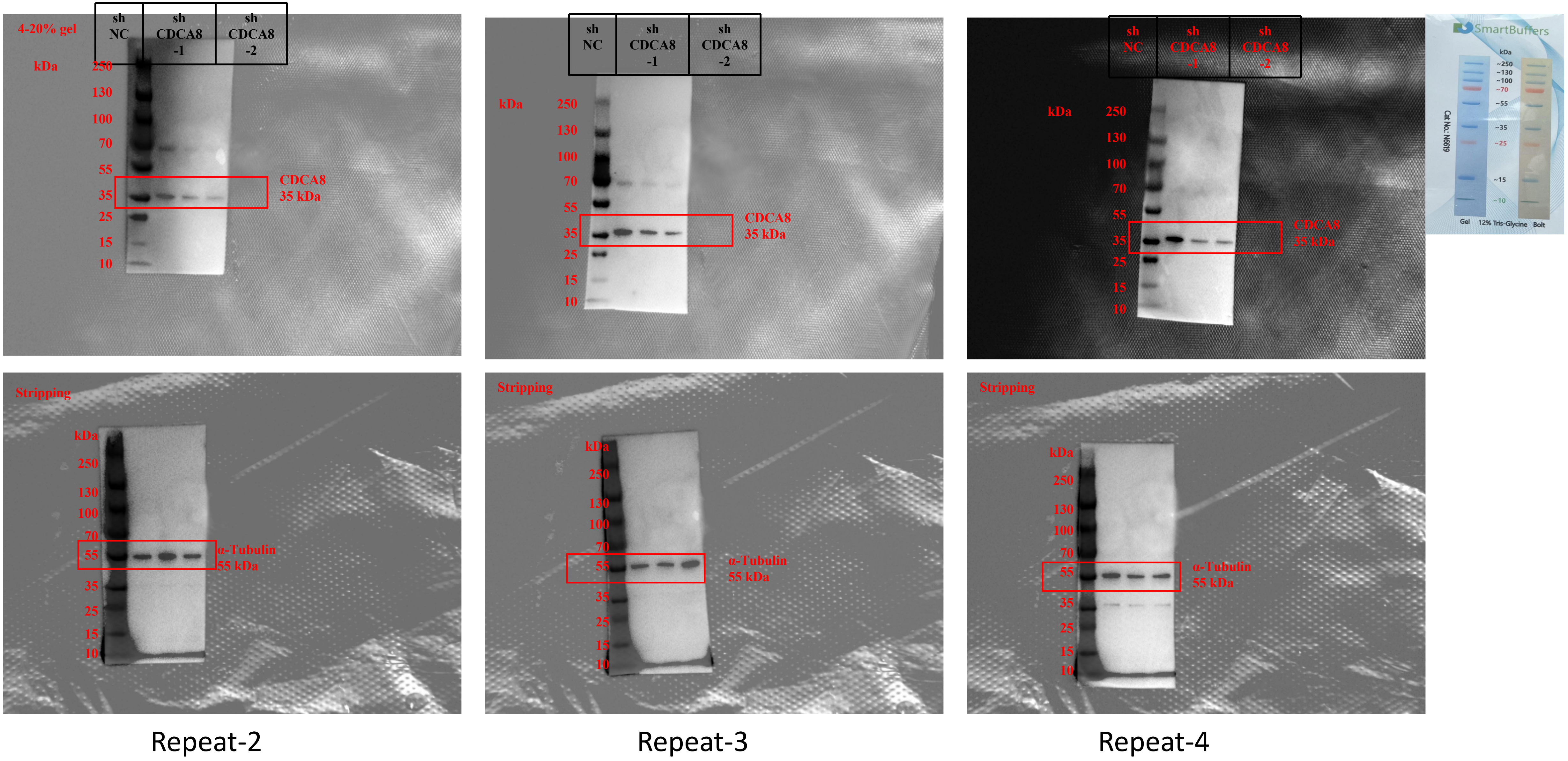

(B)WiT-49 cells were transfected with CDCA8 sh RNA or sh NC. Relative protein expression of CDCA8 was detected by Western blot.

Figure 8-B-UP. CDCA8 Regulates ATP5F1A Expression and Malignant Phenotypes in Wilms Tumor Cells-Main.

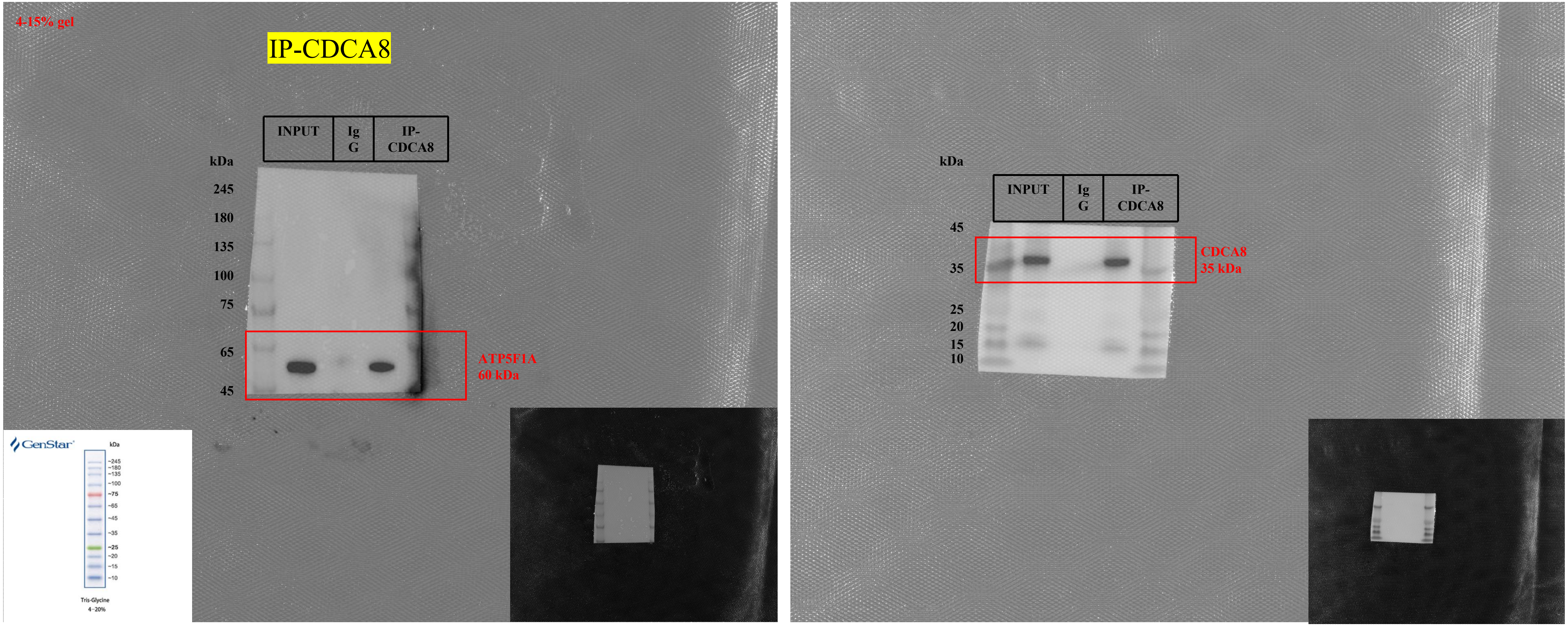

Main

(B) Co-immunoprecipitation (Co-IP) analysis confirming the specific interaction between CDCA8 and ATP5F1A in Wilms tumor cells.

Figure 8-B-UP. CDCA8 Regulates ATP5F1A Expression and Malignant Phenotypes in Wilms Tumor Cells-Repeat.

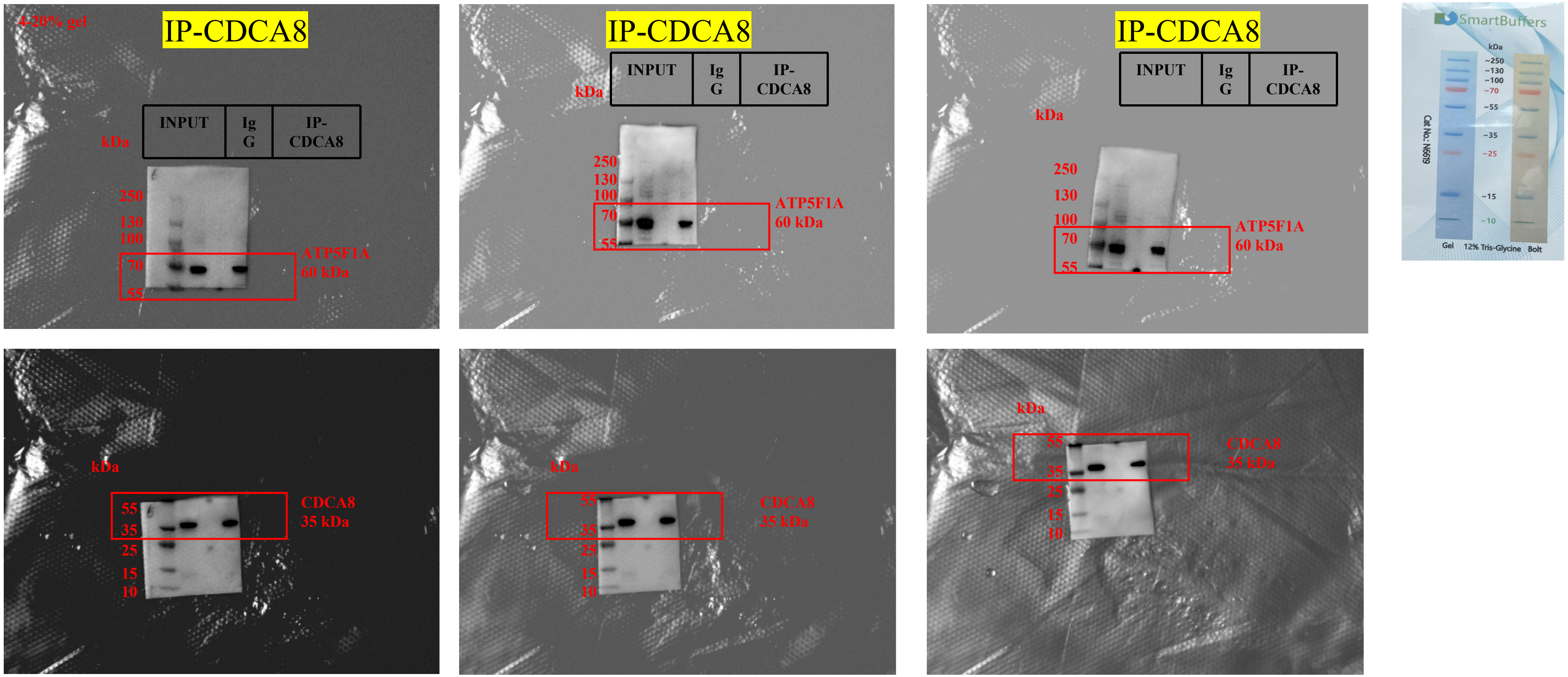

Repeat-2 Repeat-3 Repeat-4

(B) Co-immunoprecipitation (Co-IP) analysis confirming the specific interaction between CDCA8 and ATP5F1A in Wilms tumor cells.

Figure 8-B-DOWN. CDCA8 Regulates ATP5F1A Expression and Malignant Phenotypes in Wilms Tumor Cells-Main.

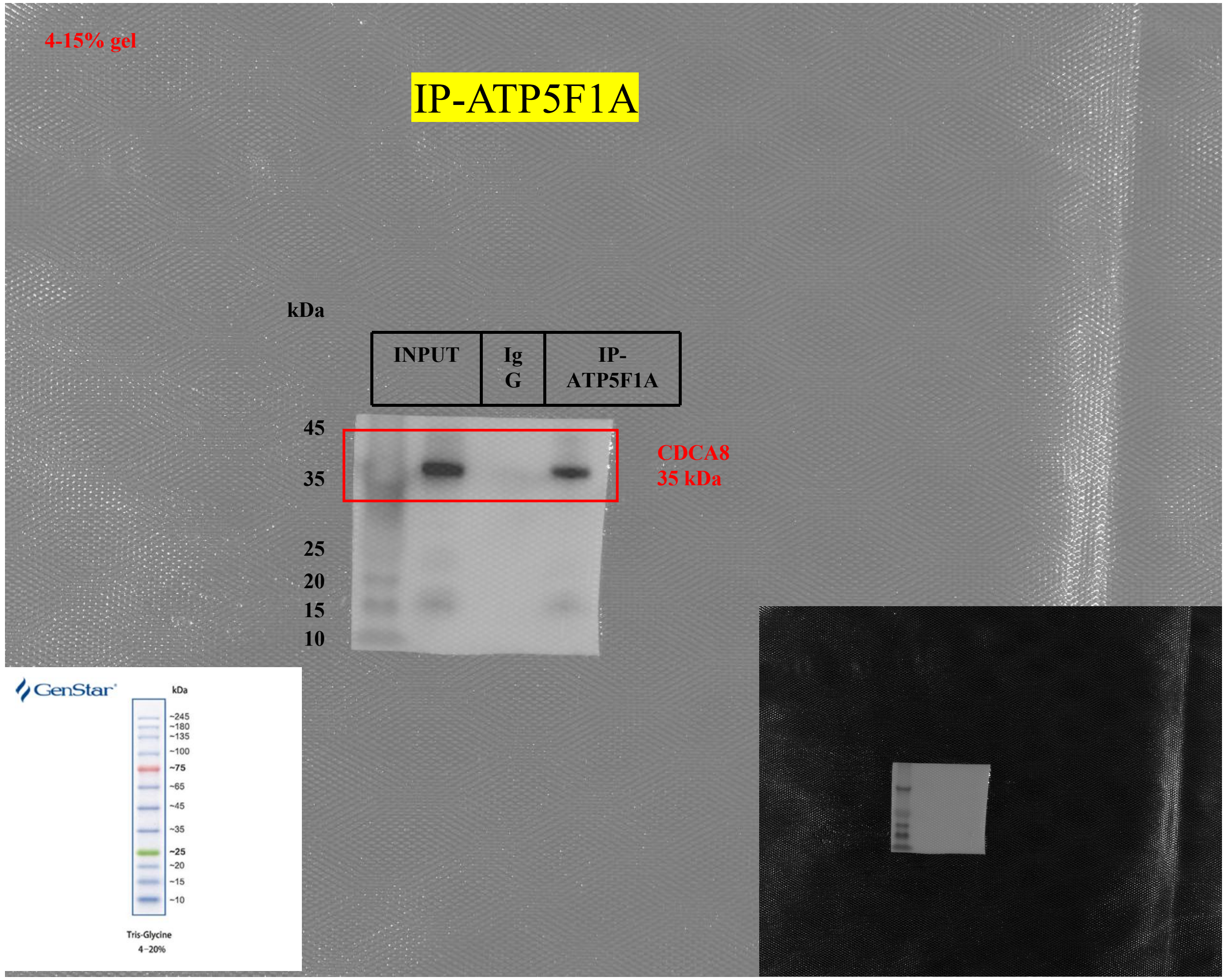

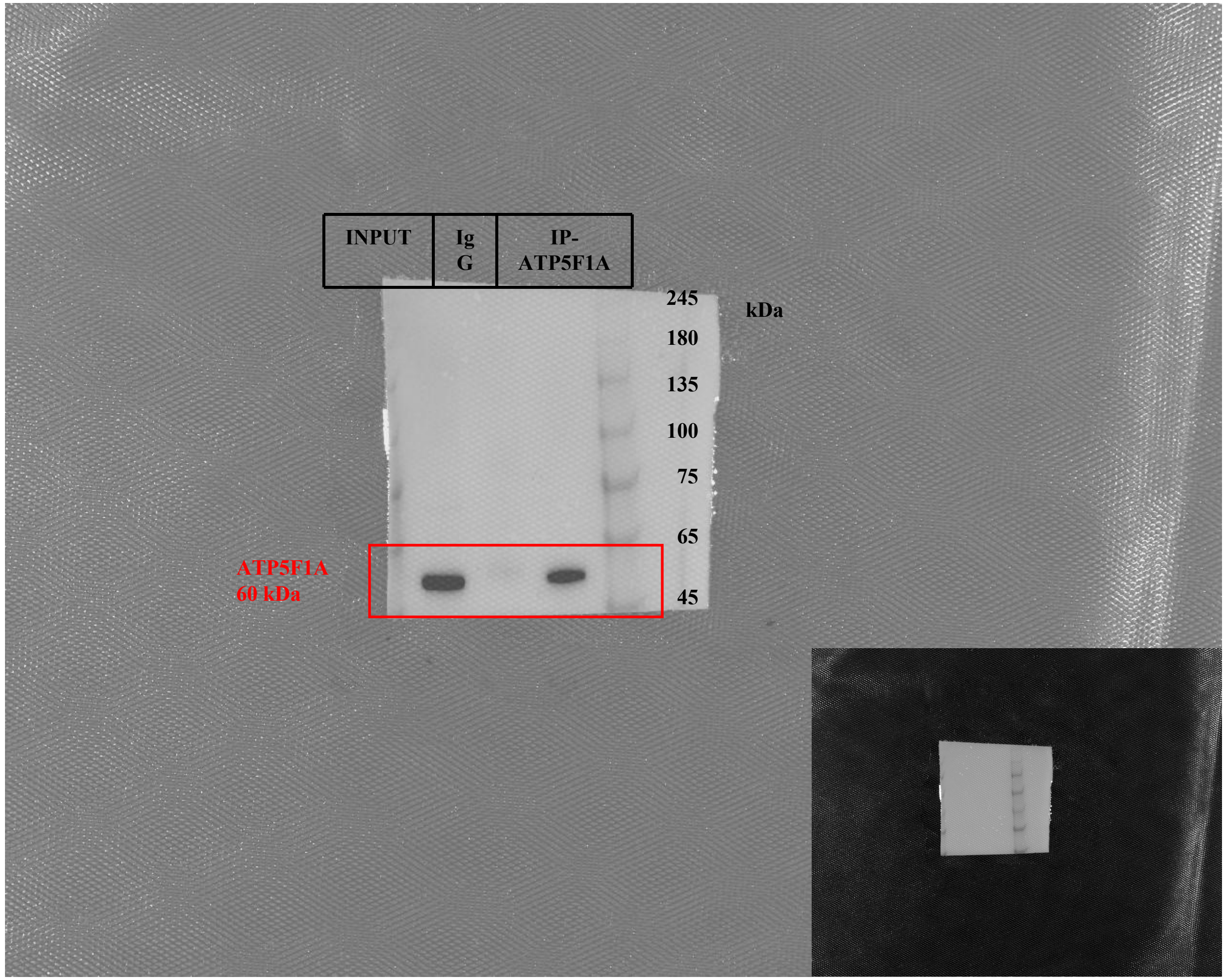

Main

(B) Co-immunoprecipitation (Co-IP) analysis confirming the specific interaction between CDCA8 and ATP5F1A in Wilms tumor cells.

Figure 8-B-DOWN. CDCA8 Regulates ATP5F1A Expression and Malignant Phenotypes in Wilms Tumor Cells-Repeat.

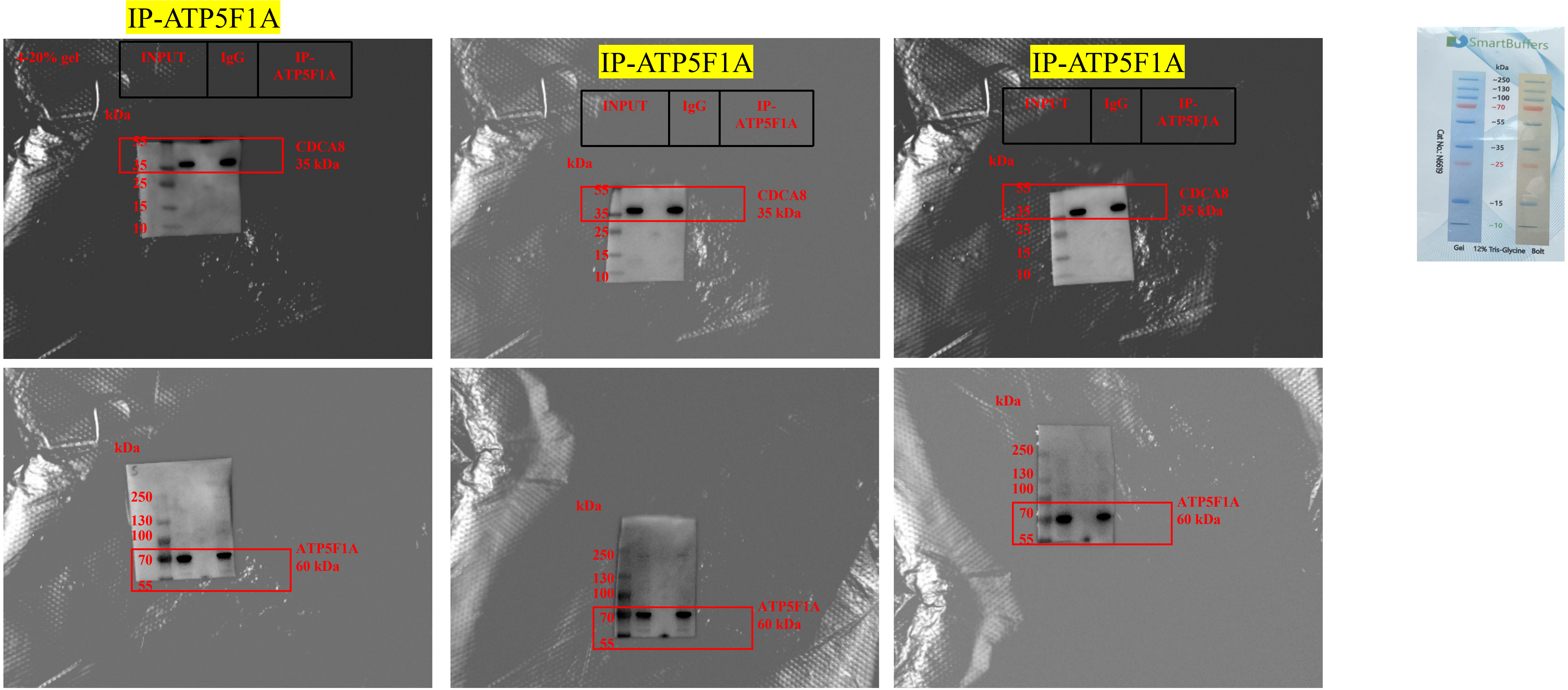

Repeat-2 Repeat-3 Repeat-4

(B) Co-immunoprecipitation (Co-IP) analysis confirming the specific interaction between CDCA8 and ATP5F1A in Wilms tumor cells.

Figure 8-D. CDCA8 Regulates ATP5F1A Expression and Malignant Phenotypes in Wilms Tumor Cells-Main.

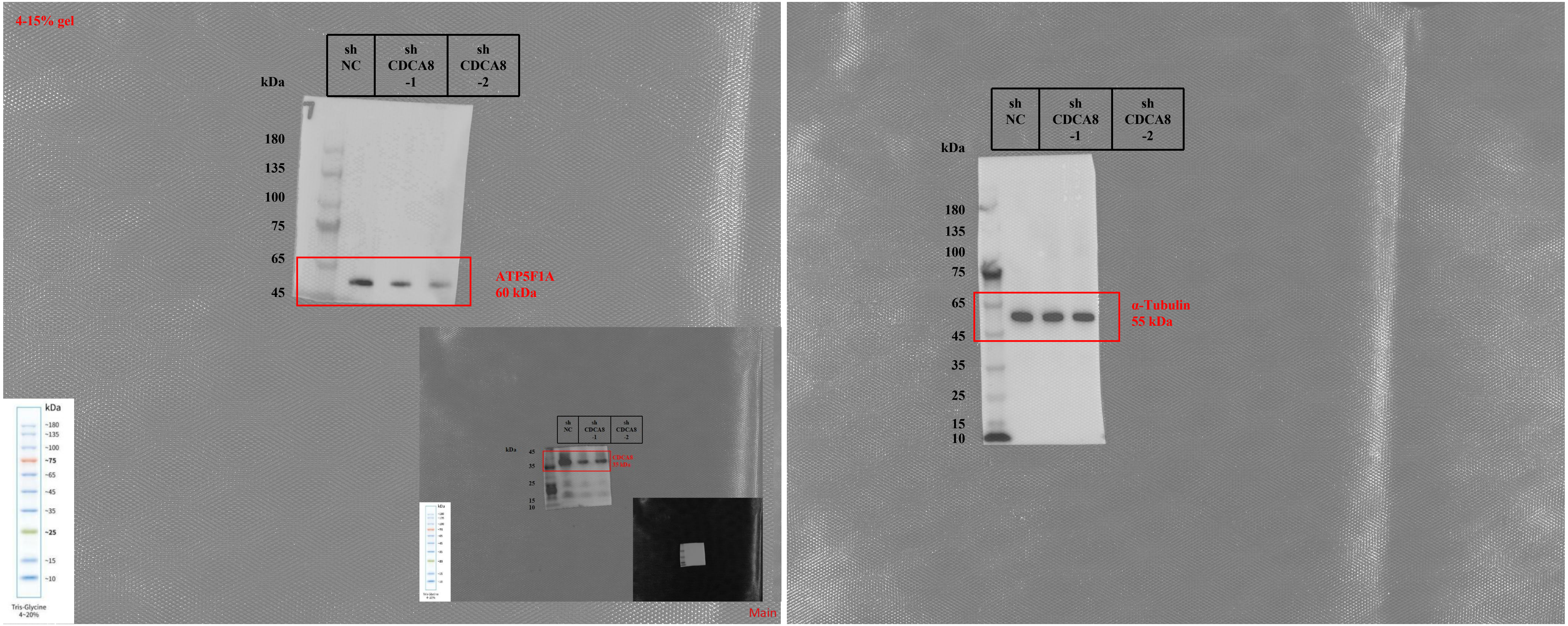

Main

(D) Protein expression analysis of ATP5F1A following CDCA8 knockdown.

Figure 8-D. CDCA8 Regulates ATP5F1A Expression and Malignant Phenotypes in Wilms Tumor Cells-Repeat.

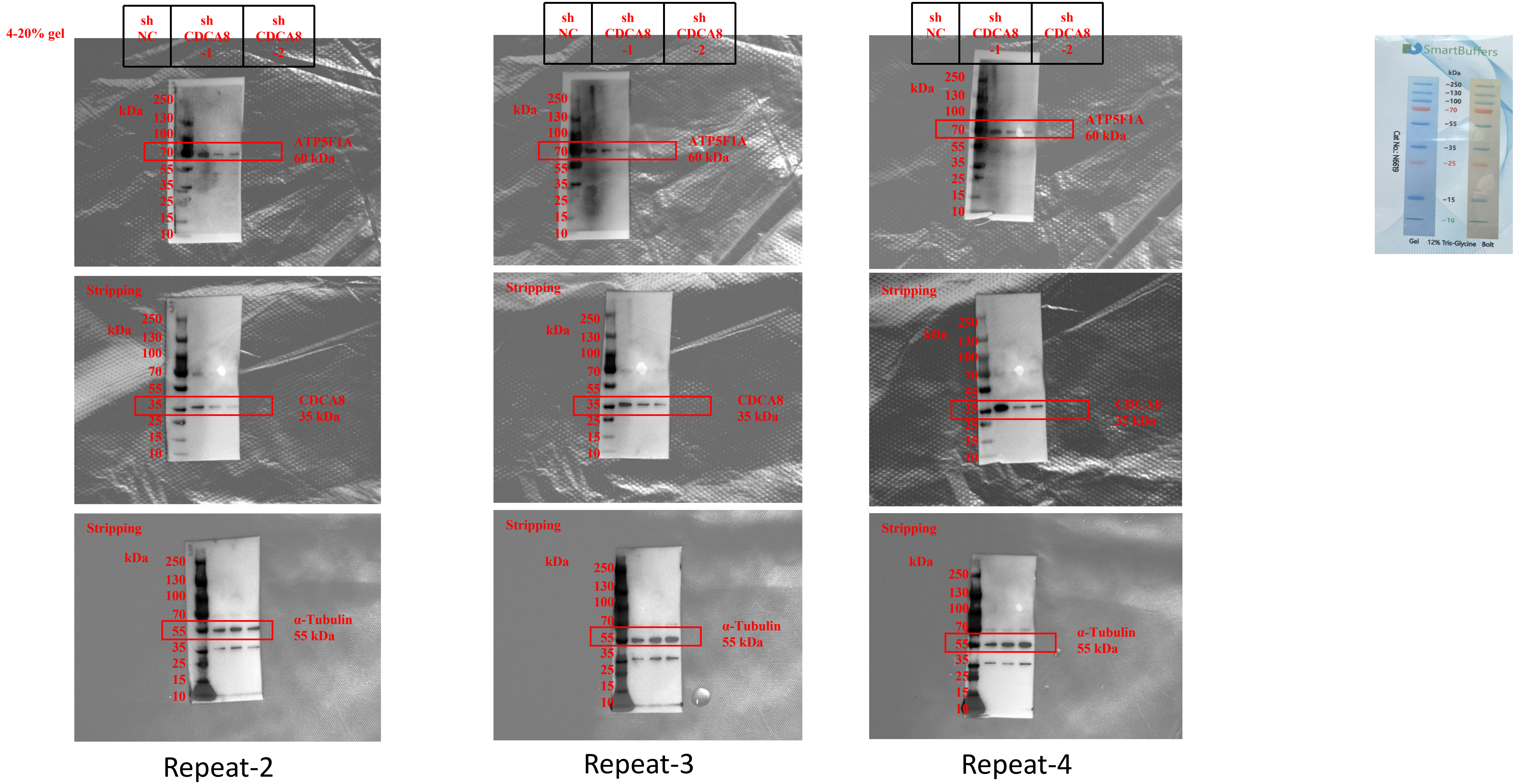

(D) Protein expression analysis of ATP5F1A following CDCA8 knockdown.

Figure 8-E. CDCA8 Regulates ATP5F1A Expression and Malignant Phenotypes in Wilms Tumor Cells-Main-Main.

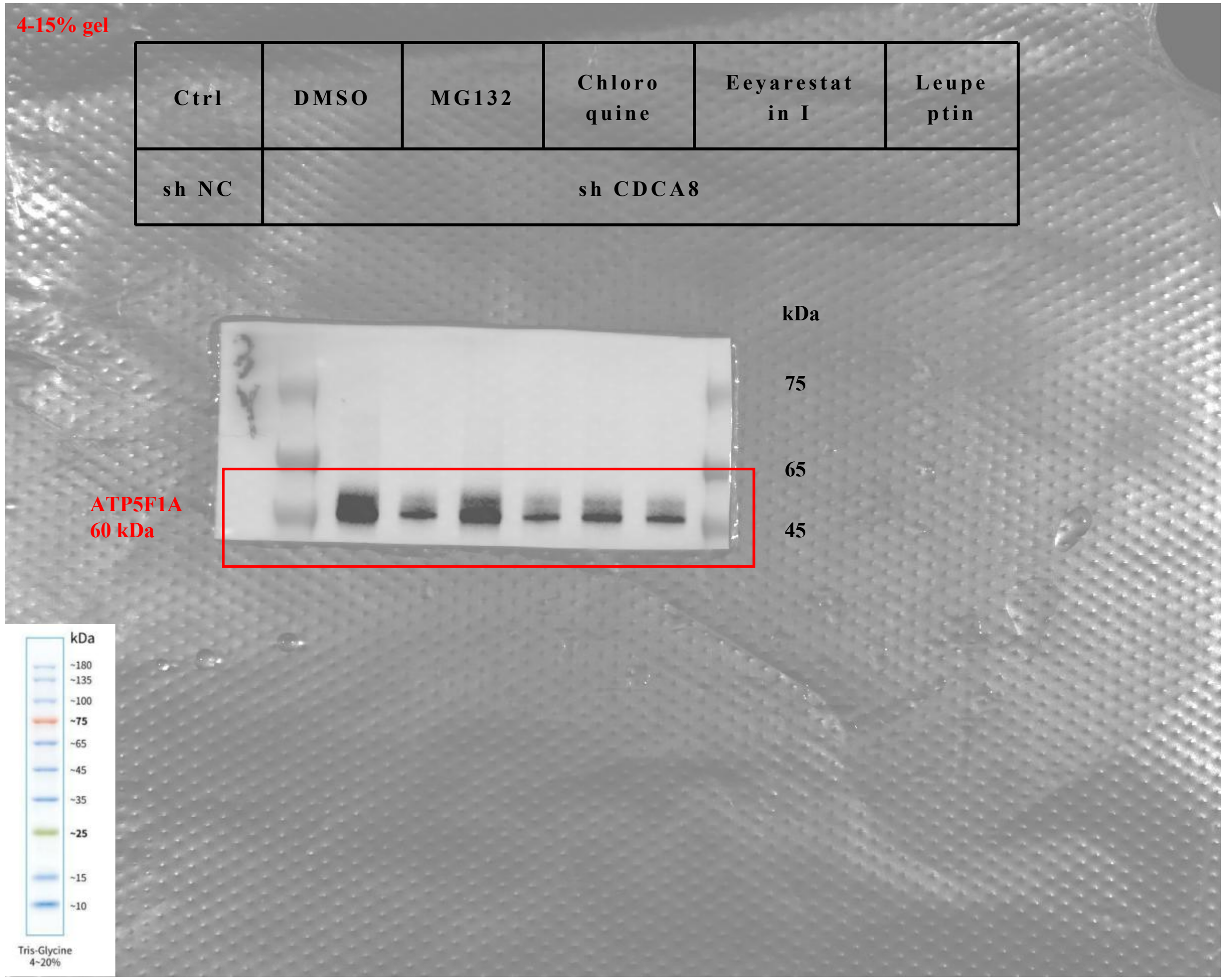

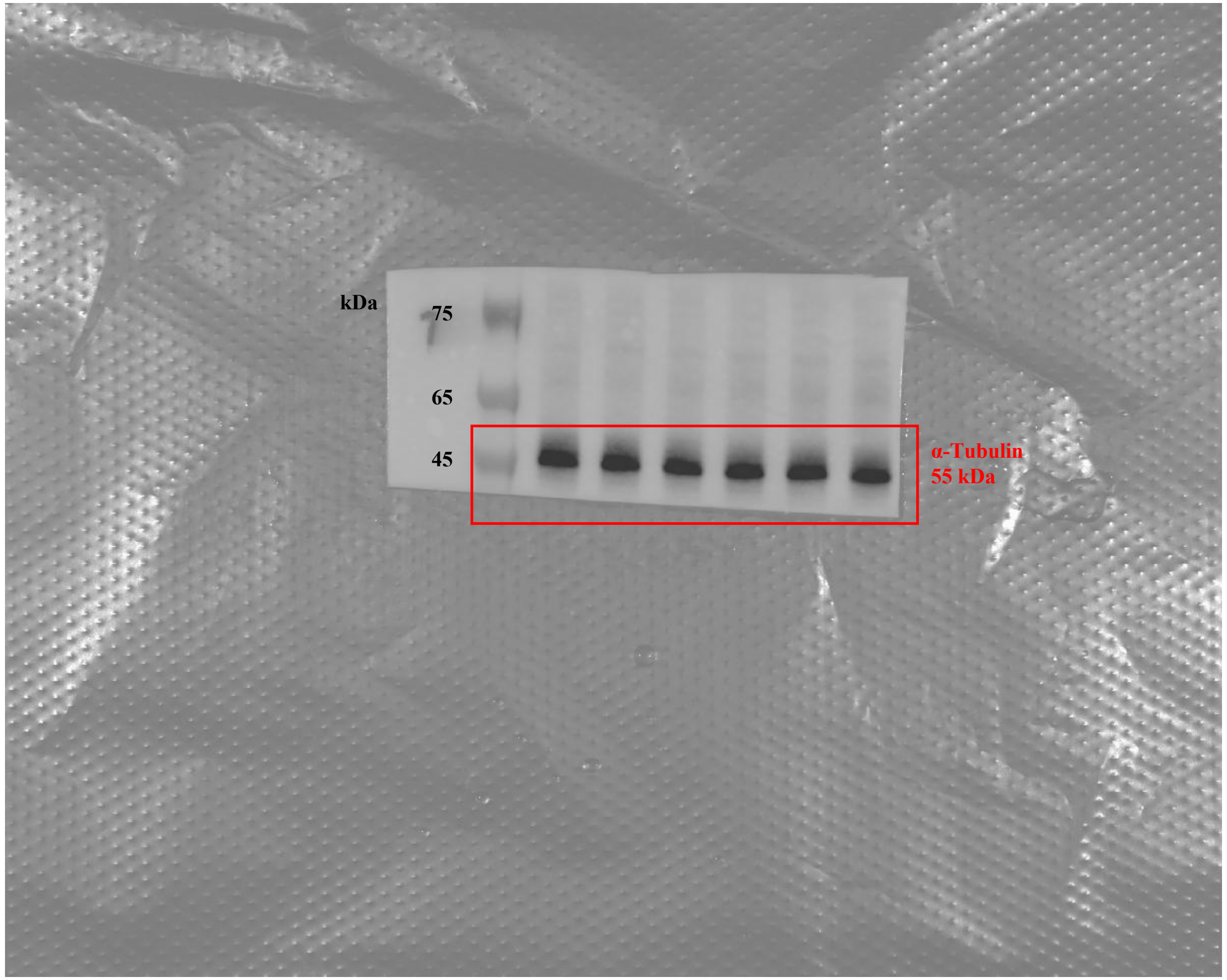

Main

(E) CDCA8 regulates ATP5F1A protein stability via the ubiquitin-proteasome system (UPS). Wilms tumor cells with CDCA8 knockdown were treated with protease inhibitors (MG132, Chloroquine, Eeyarestatin I, and Leupeptin), with MG132 significantly preventing the degradation of ATP5F1A protein.

Figure 8-E. CDCA8 Regulates ATP5F1A Expression and Malignant Phenotypes in Wilms Tumor Cells-Repeat.

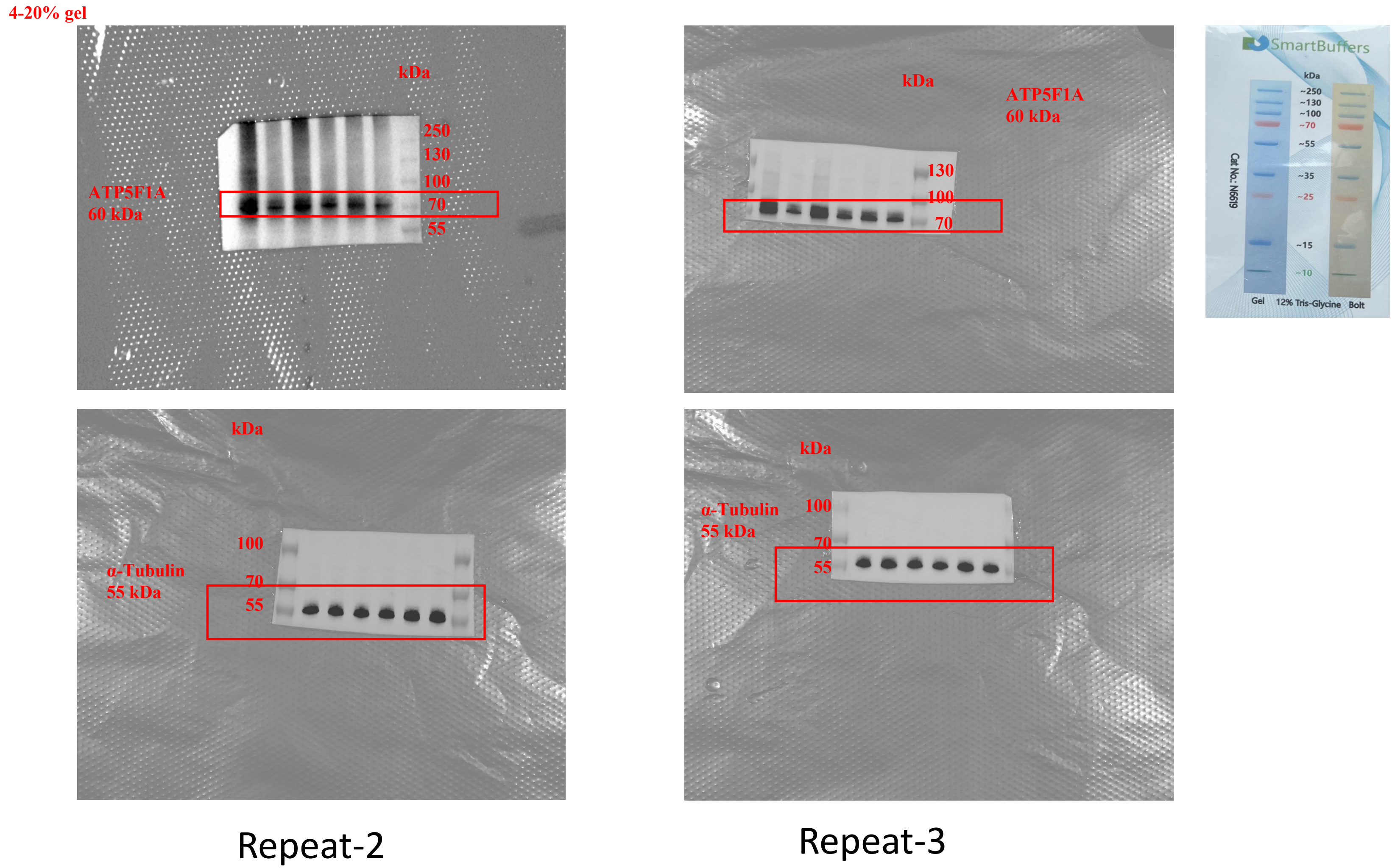

(E) CDCA8 regulates ATP5F1A protein stability via the ubiquitin-proteasome system (UPS). Wilms tumor cells with CDCA8 knockdown were treated with protease inhibitors (MG132, Chloroquine, Eeyarestatin I, and Leupeptin), with MG132 significantly preventing the degradation of ATP5F1A protein.

Figure 8-F. CDCA8 Regulates ATP5F1A Expression and Malignant Phenotypes in Wilms Tumor Cells-Main.

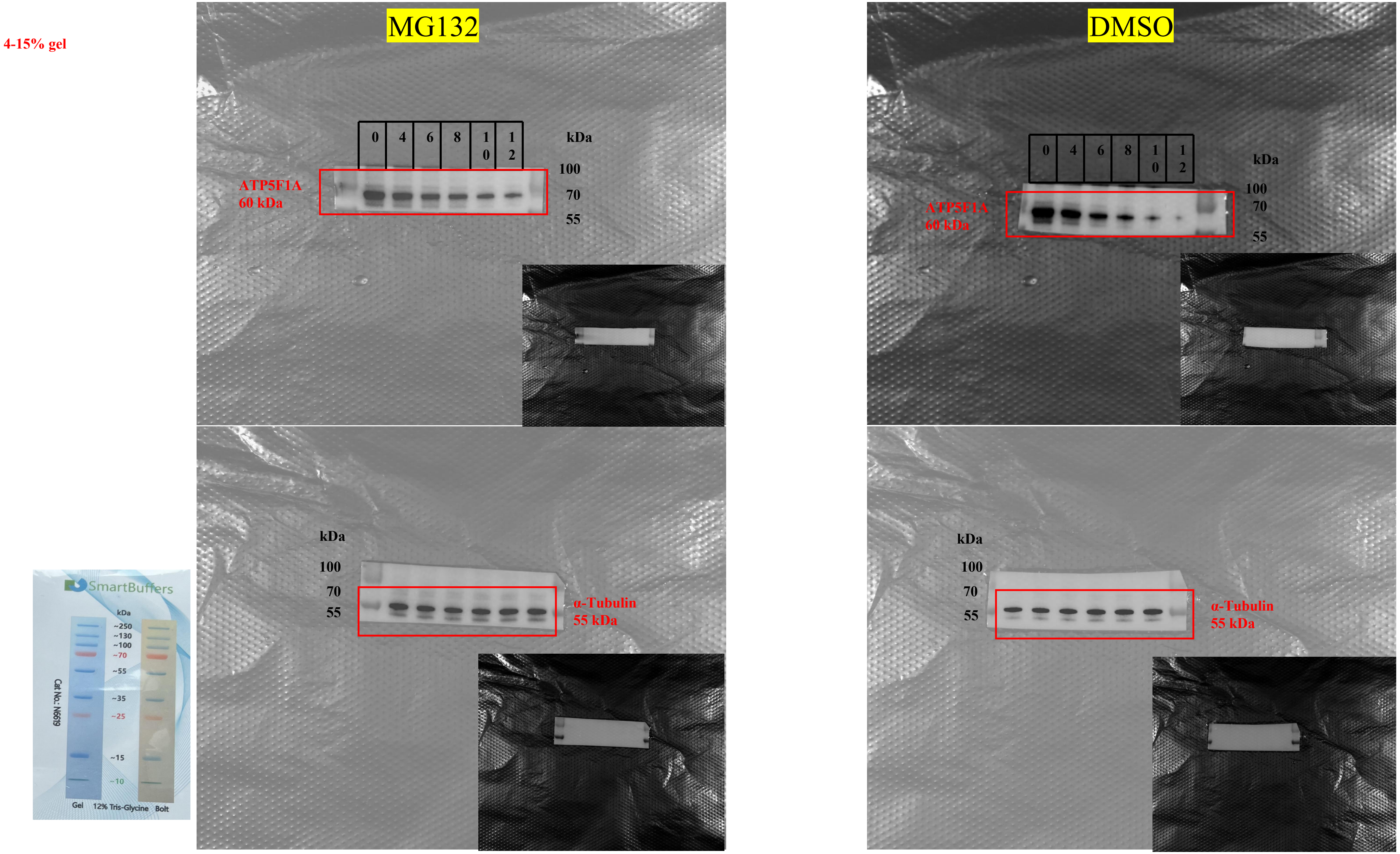

(F) The half-life of ATP5F1A is prolonged in CDCA8 knockdown cells when treated with the proteasome inhibitor MG132, indicating that CDCA8 promotes ATP5F1A degradation through the UPS.

Figure 8-F. CDCA8 Regulates ATP5F1A Expression and Malignant Phenotypes in Wilms Tumor Cells-Repeat.

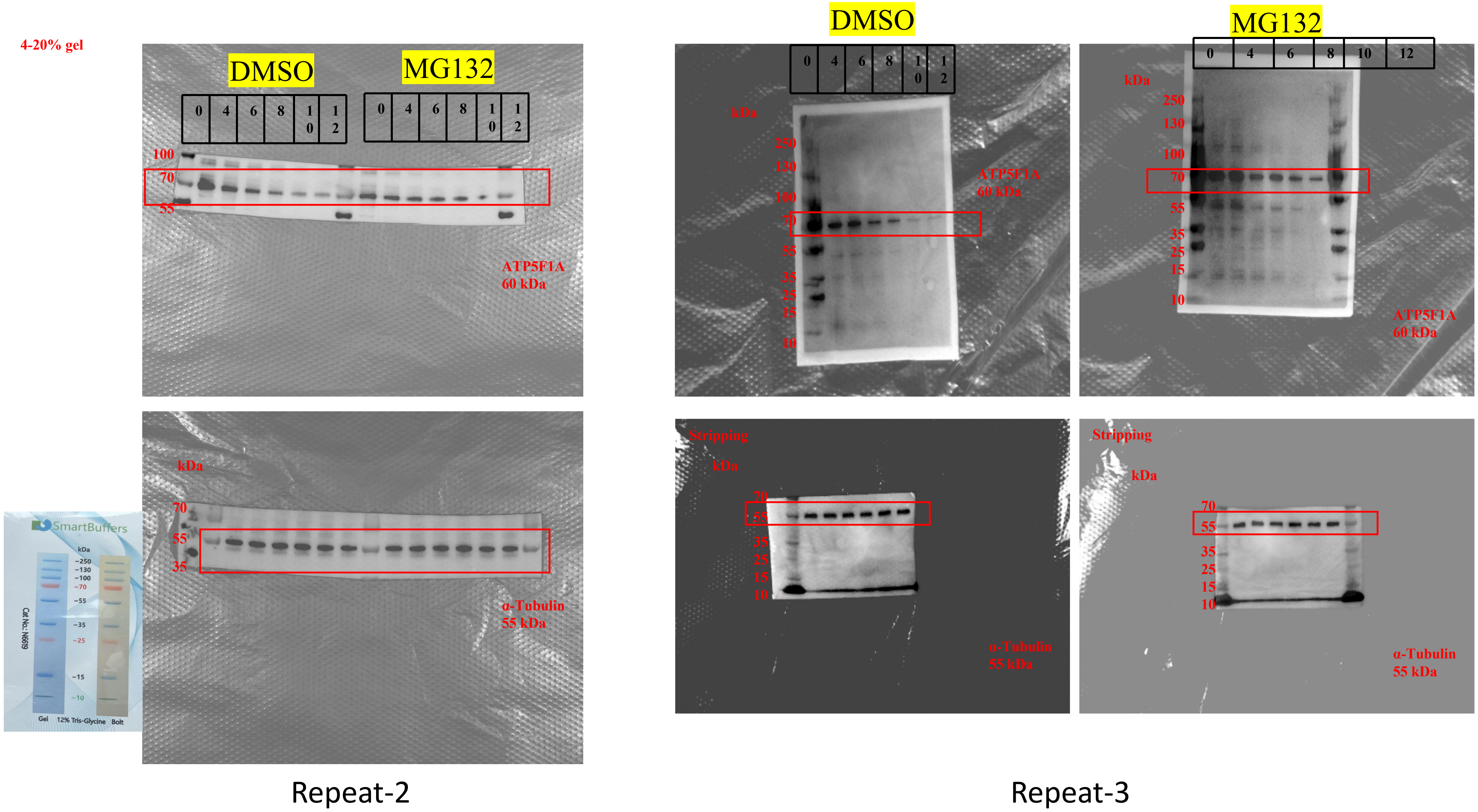

(F) The half-life of ATP5F1A is prolonged in CDCA8 knockdown cells when treated with the proteasome inhibitor MG132, indicating that CDCA8 promotes ATP5F1A degradation through the UPS.

Figure 8-G. CDCA8 Regulates ATP5F1A Expression and Malignant Phenotypes in Wilms Tumor Cells-Main.

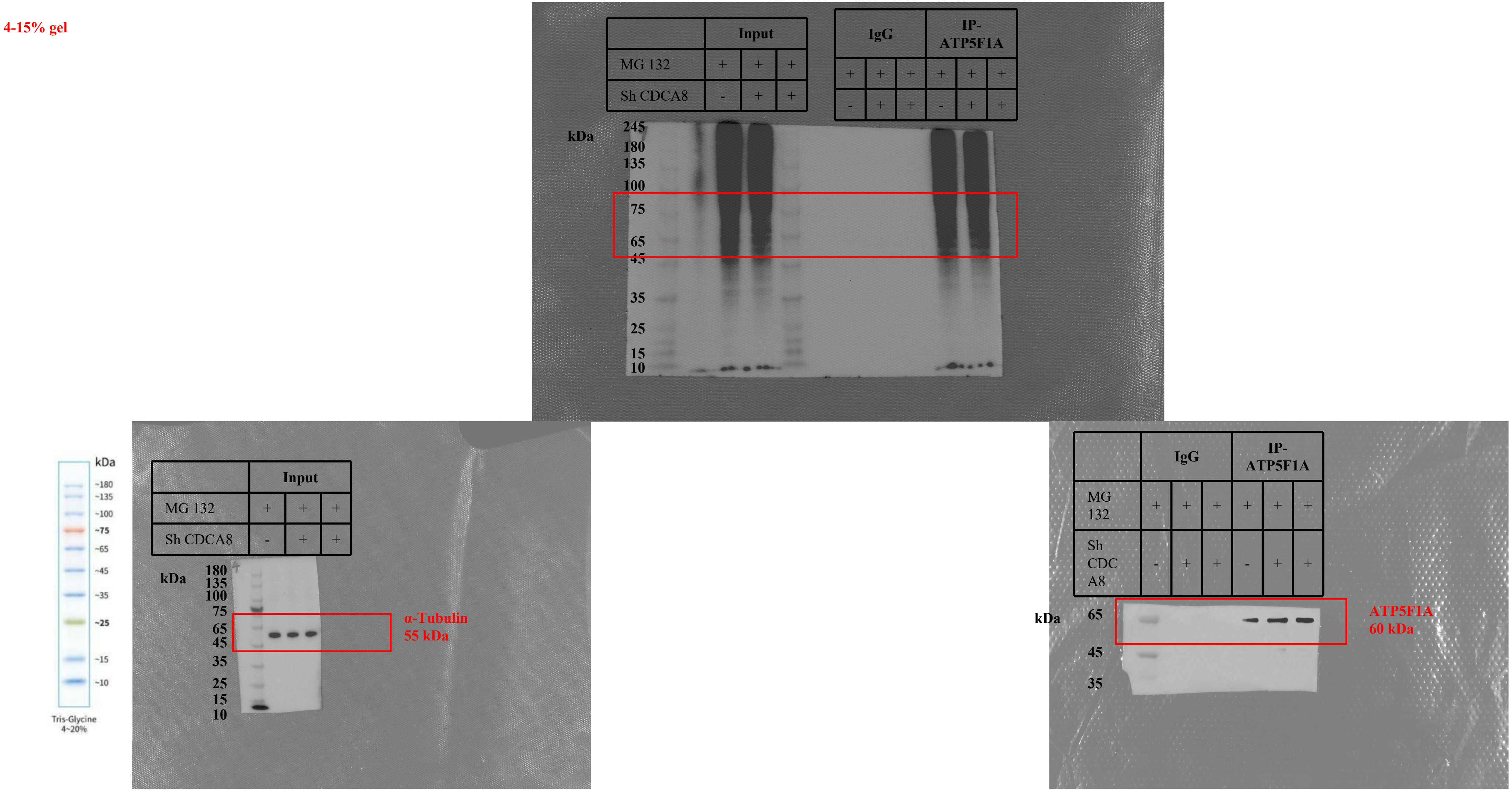

(G) Immunoprecipitation and Western blot analysis to assess ubiquitination levels of ATP5F1A in CDCA8 knockdown and control cells. Increased ubiquitination of ATP5F1A is observed in CDCA8 knockdown cells, supporting the role of CDCA8 in promoting ATP5F1A ubiquitination and proteasomal degradation.

Figure 8-G. CDCA8 Regulates ATP5F1A Expression and Malignant Phenotypes in Wilms Tumor Cells-Repeat.

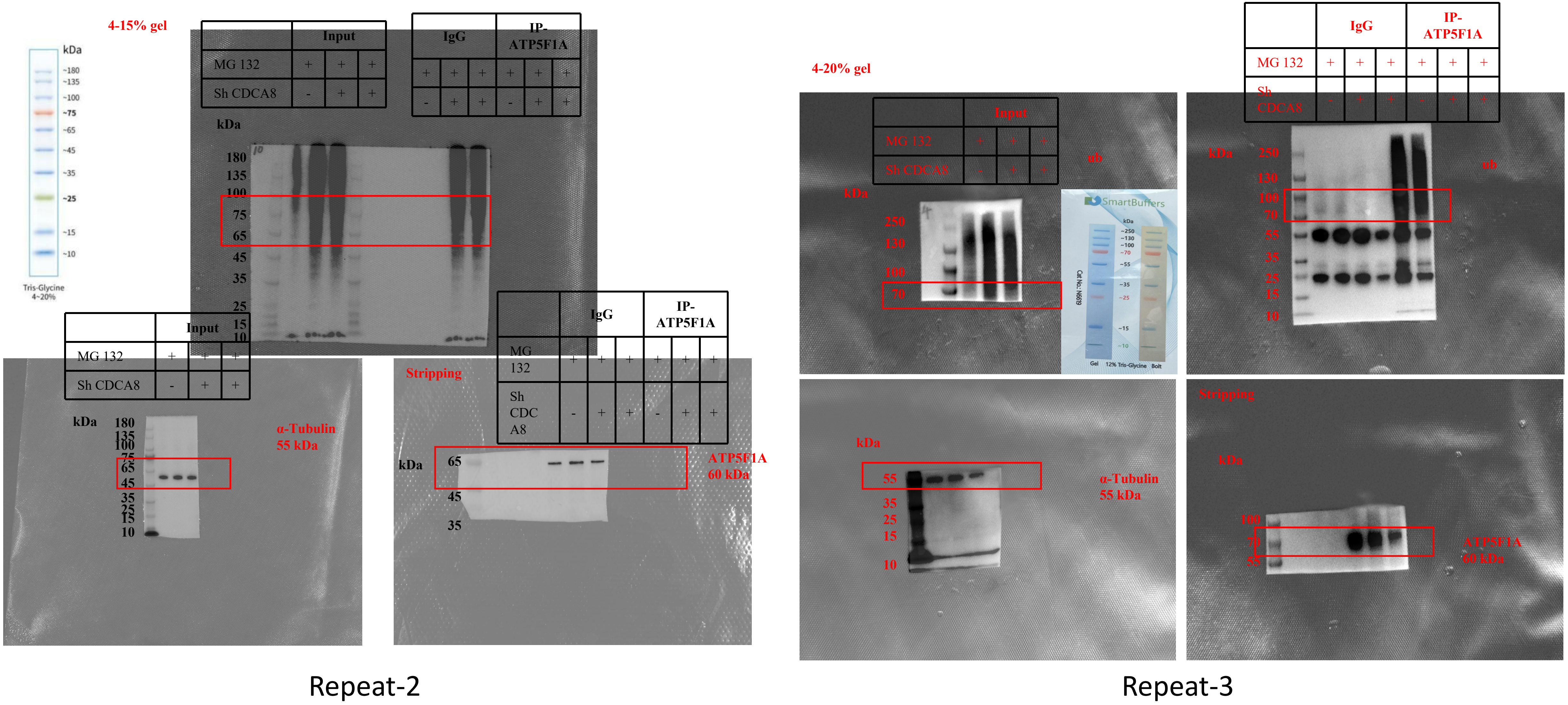

(G) Immunoprecipitation and Western blot analysis to assess ubiquitination levels of ATP5F1A in CDCA8 knockdown and control cells. Increased ubiquitination of ATP5F1A is observed in CDCA8 knockdown cells, supporting the role of CDCA8 in promoting ATP5F1A ubiquitination and proteasomal degradation.

Figure 8-H. CDCA8 Regulates ATP5F1A Expression and Malignant Phenotypes in Wilms Tumor Cells-Main.

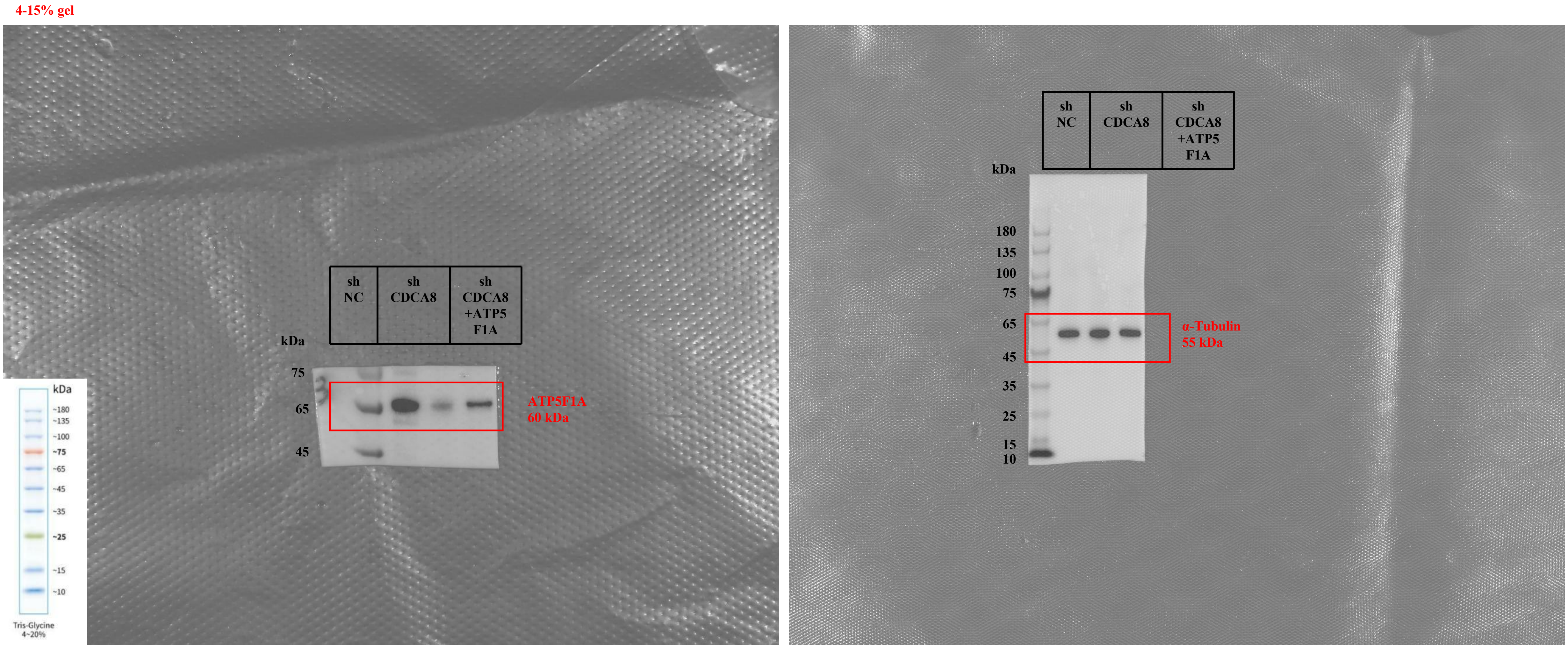

(H) ATP5F1A overexpression rescue experiment in CDCA8-knockdown Wilms tumor cells to evaluate the restoration of malignant phenotypes.

Figure 8-H. CDCA8 Regulates ATP5F1A Expression and Malignant Phenotypes in Wilms Tumor Cells-Repeat.

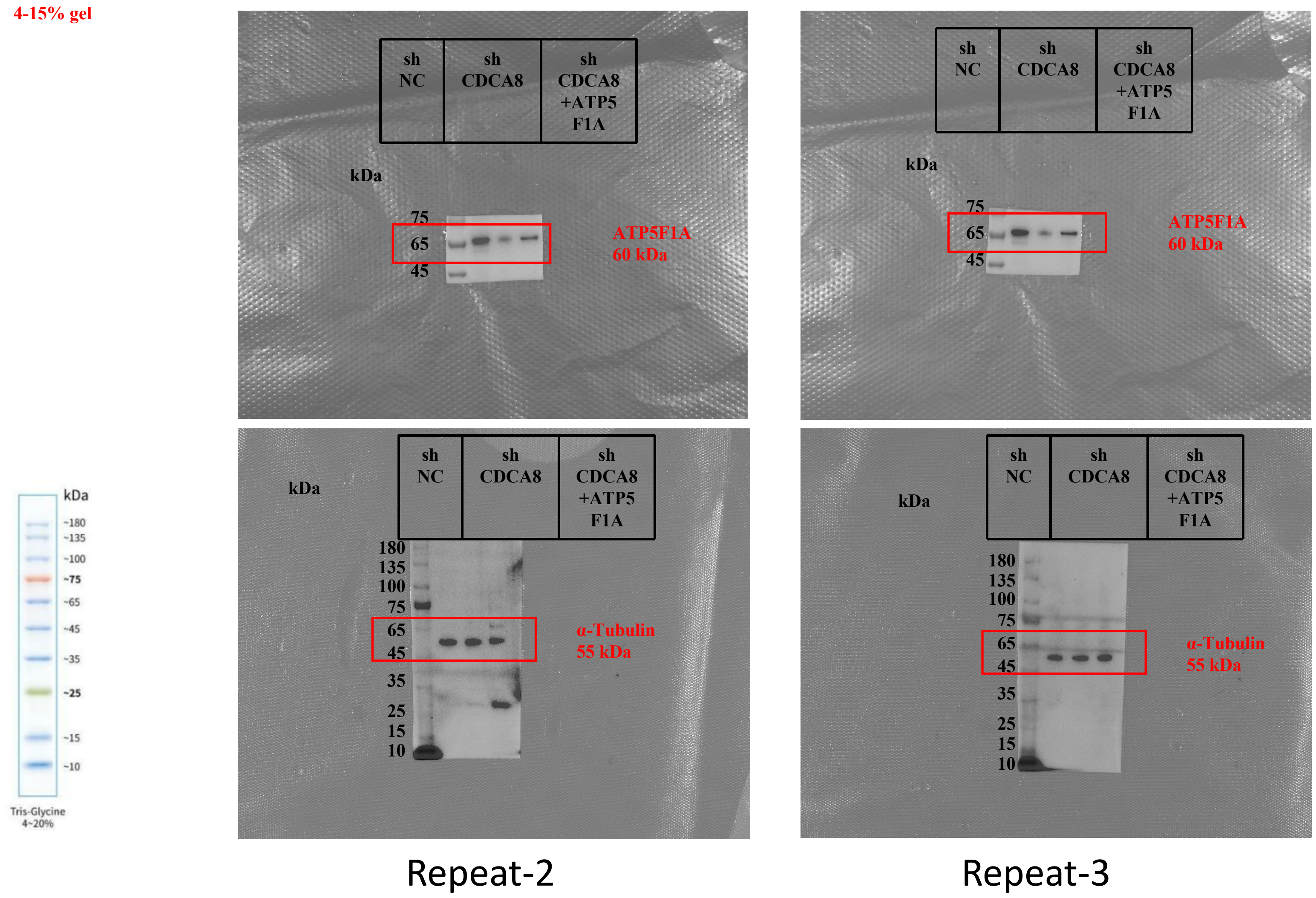

(H) ATP5F1A overexpression rescue experiment in CDCA8-knockdown Wilms tumor cells to evaluate the restoration of malignant phenotypes.
